# Causal effects of gut microbiota on sepsis: a two-sample Mendelian randomization study

**DOI:** 10.3389/fmicb.2023.1167416

**Published:** 2023-05-10

**Authors:** Jie-Hai Chen, Li-Ying Zeng, Yun-Feng Zhao, Hao-Xuan Tang, Hang Lei, Yu-Fei Wan, Yong-Qiang Deng, Ke-Xuan Liu

**Affiliations:** ^1^Department of Anesthesiology, Nanfang Hospital, Southern Medical University, Guangzhou, Guangdong, China; ^2^Guangdong Provincial Key Laboratory of Proteomics, Department of Pathophysiology, School of Basic Medical Sciences, Southern Medical University, Guangzhou, Guangdong, China

**Keywords:** Mendelian randomization, gut microbiota, sepsis, causal inference, genetics

## Abstract

**Background:**

Recent studies had provided evidence that the gut microbiota is associated with sepsis. However, the potential causal relationship remained unclear.

**Methods:**

The present study aimed to explore the causal effects between gut microbiota and sepsis by performing Mendelian randomization (MR) analysis utilizing publicly accessible genome-wide association study (GWAS) summary-level data. Gut microbiota GWAS (*N* = 18,340) were obtained from the MiBioGen study and GWAS-summary-level data for sepsis were gained from the UK Biobank (sepsis, 10,154 cases; 452,764 controls). Two strategies were used to select genetic variants, i.e., single nucleotide polymorphisms (SNPs) below the locus-wide significance level (1 × 10^−5^) and the genome-wide statistical significance threshold (5 × 10^−8^) were chosen as instrumental variables (IVs). The inverse variance weighted (IVW) was used as the primary method for MR study, supplemented by a series of other methods. Additionally, a set of sensitivity analysis methods, including the MR-Egger intercept test, Mendelian randomized polymorphism residual and outlier (MR-PRESSO) test, Cochran’s Q test, and leave-one-out test, were carried out to assess the robustness of our findings.

**Results:**

Our study suggested that increased abundance of *Deltaproteobacteria, Desulfovibrionales, Catenibacterium*, and *Hungatella* were negatively associated with sepsis risk, while *Clostridiaceae1, Alloprevotella, LachnospiraceaeND3007group*, and *Terrisporobacter* were positively correlated with the risk of sepsis. Sensitivity analysis revealed no evidence of heterogeneity and pleiotropy.

**Conclusion:**

This study firstly found suggestive evidence of beneficial or detrimental causal associations of gut microbiota on sepsis risk by applying MR approach, which may provide valuable insights into the pathogenesis of microbiota-mediated sepsis and strategies for sepsis prevention and treatment.

## 1. Background

Sepsis is defined as life-threatening organ dysfunction arising from dysregulation of the host inflammatory and immune response to infection (Singer et al., 2016). It is regarded as a major cause of health loss and a major contributor to the global burden of disease. In 2017 alone, an estimated 49 million cases of sepsis occurred globally and there were 11 million sepsis-related deaths, accounting for 20% of all deaths that year worldwide (Rudd et al., 2020). A global epidemiological data shows that intensive care unit (ICU) and hospital mortality rates for patients with sepsis are 26 and 35%, respectively (Vincent et al., 2014). Despite the implementation of early goal-directed therapy and individualized treatment strategies for sepsis, which remains one of the leading causes of morbidity and mortality worldwide, and the World Health Organization has recognized sepsis as a global health priority (Reinhart et al., 2017). Searching for therapeutic or prophylactic targets against sepsis and improving sepsis outcomes is a pressing need.

Gut microbiota modulates the physiological homeostasis of the host, including intestinal barrier function, immune system, and disease vulnerability pathways (Kamada et al., 2013). The role of gut microbiota in sepsis has fueled growing interest among researchers. With the development and application of technologies such as 16S rRNA and metagenomic sequencing (Zoetendal et al., 2008), there is mounting proof that gut microbiota plays a significant role in the pathophysiology of sepsis. Several observational studies showed that the composition of the gut microbiota is heavily affected by sepsis, which may in turn result in organ failure (Zaborin et al., 2014; Ojima et al., 2016; Chen et al., 2022; Deng et al., 2023a,b). Another observational investigation revealed that the percentages of *Enterococcus* and *Klebsiella* were considerably higher in septic patients than in healthy controls. However, compared to healthy controls, septic patients had much lower proportions of *Faecalibacterium* and *Blautia* (Mu et al., 2022). Some pre-clinical models have demonstrated that the application of antibiotics disrupts the gut microbiome and raises the risk of bloodstream infections and critical conditions (Ubeda et al., 2010; Ayres et al., 2012). According to a study of 10,996 participants in the U.S. Health and Retirement Study, hospitalizations known to be linked to periods of microbiota perturbation were linked to a higher risk of severe sepsis after hospital discharge. Events known to cause dysbiosis and subsequent admission for severe sepsis were strongly correlated in a dose-response fashion (Prescott et al., 2015). A meta-analysis of randomized controlled trials and observational studies revealed that probiotic management is beneficial in preventing late-onset sepsis in preterm infants (Dermyshi et al., 2017).

A growing body of evidence has demonstrated an association between gut microbiota and sepsis. However, causality cannot be reliably established in conventional observational studies due to confounding and reverse causation, which can lead to biased conclusions. These limitations can be overcome by Mendelian randomization (MR), an epidemiological approach that infers exposure-outcome causation using genetic variants as instrumental variables (IVs). Single nucleotide polymorphisms (SNPs) are allocated randomly at conception and are independent of confounding factors, making MR comparable to randomized controlled trials and circumventing the drawbacks of previous observational studies (Lawlor et al., 2008; Davey and Hemani, 2014; Davies et al., 2018; Richmond and Davey, 2022). To date, no studies have assessed the role of gut microbiota on the risk of sepsis in the framework of MR. Therefore, the present study first employed a two-sample MR methodology to explore causality between genetically predicted gut microbiota and sepsis risk by leveraging summary statistics from large genome-wide association studies (GWAS).

## 2. Methods

### 2.1. Study design and data sources

**Figure 1:**
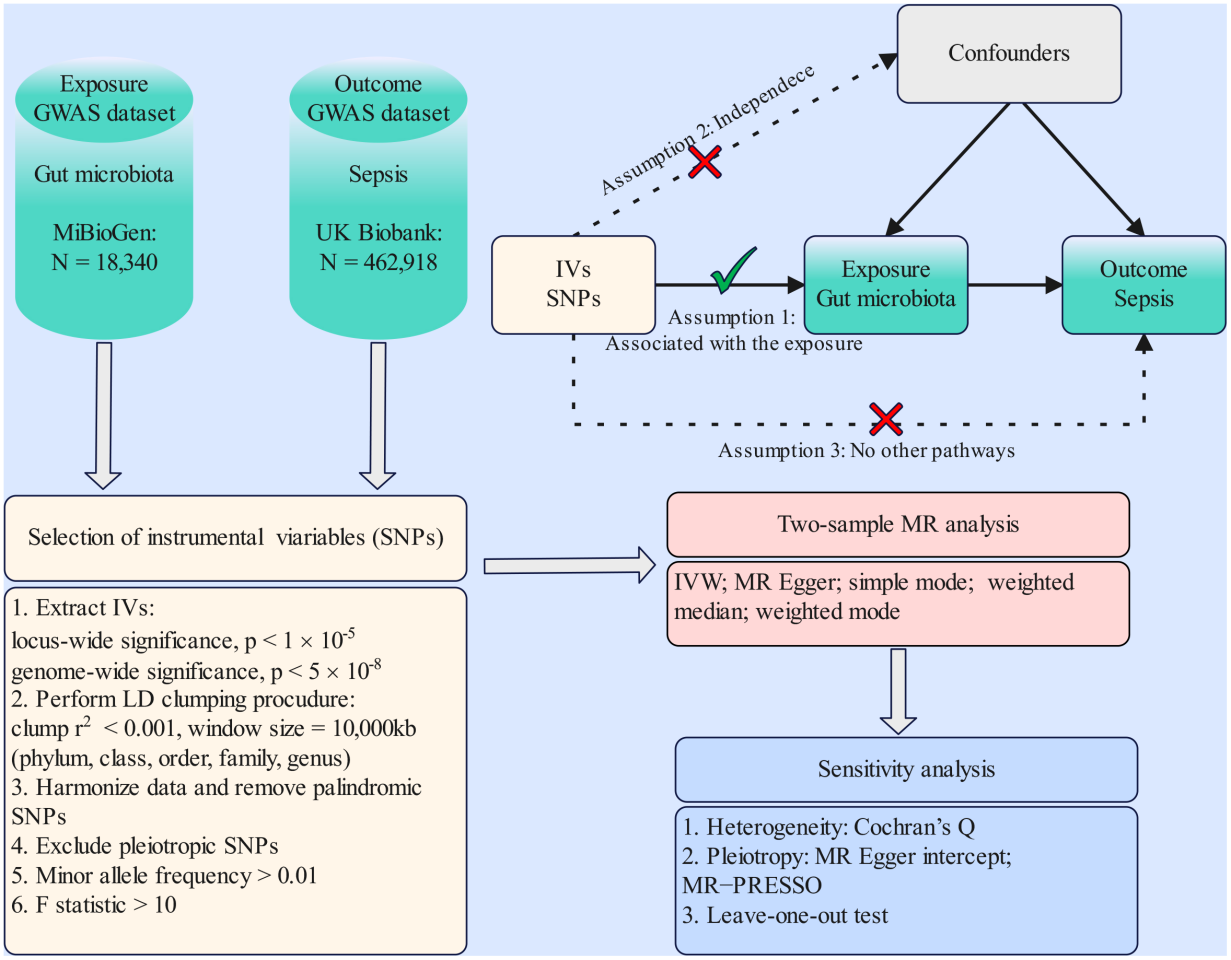
Flowchart of the present MR study and major assumptions. MR, Mendelian randomization; GWAS, genome-wide association study; SNPs, single-nucleotide polymorphisms; IVW, inverse-variance weighted; LD, linkage disequilibrium; MR-PRESSO, MR pleiotropy residual sum and outlier.

MR study was applied to investigate the causal effects between gut microbiota and sepsis. The flowchart of our work is presented in Figure 1. In brief, genetic variants associated with the exposure were extracted from GWAS summary statistics and served as IVs. Two-sample MR analysis involving five MR methods was carried out sequentially. Ultimately, a set of sensitivity analysis measures including the heterogeneity test, pleiotropy test, and leave-one-out test were performed for significant associations.

GWAS summary-level data of gut microbiota were obtained from the MiBioGen study (Kurilshikov et al., 2021; Mibiogen consortium, 2022). It was the largest, multiracial, genome-wide meta-analysis of the gut microbiota to date, analyzing genome-wide genotyping data and 16S fecal microbiota data from 24 cohorts (18,340 individuals). Most of the participants surveyed were of European descent (*N* = 13,266). Targeting the V4, V3-V4, and V1-V2 regions of the 16S rRNA gene allowed the microbial composition to be profiled. Direct taxonomic binning was then used to undertake a taxonomic classification. After 16S microbiome data processing, 211 taxa involving 131 genera, 35 families, 20 orders, 16 classes, and 9 phyla were finally identified. Relevant details about the microbiota data were reported in the original study (Kurilshikov et al., 2021). Summary-level data of GWAS for sepsis were generated from the UK Biobank, which included 10,154 sepsis cases and 452,764 controls (Ponsford et al., 2020). Women constitute 54% and men represent 46%. The median age of all participants was 58 years, and the median age of sepsis cases was 60 years. Sepsis is defined by a published list of definitive International Classification of Disease-9 and International Classification of Disease-10 codes derived by a panel of experts in critical care, infectious diseases, pediatrics, and sepsis epidemiology (Rudd et al., 2020). More details on the sepsis GWAS were described elsewhere (Ponsford et al., 2020).

### 2.2. Instrumental variables selection

Bacterial taxa were categorized and analyzed at five taxonomic levels (phylum, class, order, family, and genus). To guarantee the accuracy and validity of conclusions about the causality between gut microbiota and sepsis risk, the following quality control procedures were applied to filter IVs. First, single-nucleotide polymorphisms (SNPs) with a significant relationship to the gut microbiome were chosen as IVs. SNPs were selected using two thresholds. To obtain more comprehensive results and increase the explained phenotypic variance, a series of SNPs with lower than locus-wide significance level (1 × 10^−5^) were picked as IVs. Another set of SNPs at genome-wide significance (*p* < 5 × 10^−8^) were chosen as IVs for the secondary analysis. Second, to ensure independence among the selected IVs and to minimize the effect of linkage disequilibrium that violates random allele assignment, the parameters of the clumping procedure were set to *r*^2^ < 0.001 and kb = 10,000 kb. Third, the proxy SNPs strongly correlated to the target variant (*r*^2^ > 0.8) were chosen when exposure-related SNPs were missing from the outcome GWAS by searching the SNiPA website^1^ (Arnold et al., 2015). For example, rs9813669 for rs9833771, rs62115123 for rs75211493, rs809311 for rs789068, more information see Supplementary Table S3. Fourth, SNPs for being palindromic and incompatible alleles were disqualified from the MR. Fifth, in order to satisfy the second key assumption (independent of the confounders), i.e., IVs are not significantly correlated with confounding factors, we manually checked and excluded SNPs that were significantly associated (*p* < 5 × 10^−8^) with confounders through the PhenoScanner GWAS database^2^ (Kamat et al., 2019). SNPs rs1530559 and rs182549 were eliminated because they were closely (*p* < 5 × 10^−8^) associated with total cholesterol and body mass index, respectively. The SNP rs12636310 was removed because it was linked to two potential confounders, type II diabetes and body mass index (*p* < 5 × 10^−8^) (Mohus et al., 2022). Sixth, the minor allele frequency must be above 0.01. Lastly, to avoid weak instrumental bias, the F-statistic (Burgess and Thompson, 2011) for each SNP was determined, and SNPs with F-statistics lower than 10 were discarded, if any. The F-statistic is expressed as *R*^2^*(n-k-*1*)/k(*1*-R*^2^*)*. In the formula, *n, k,* and *R*^2^ represent the sample size, the number of IVs and the variance interpreted by the IVs, correspondingly.

### 2.3. Effect size estimate

**Figure 2:**
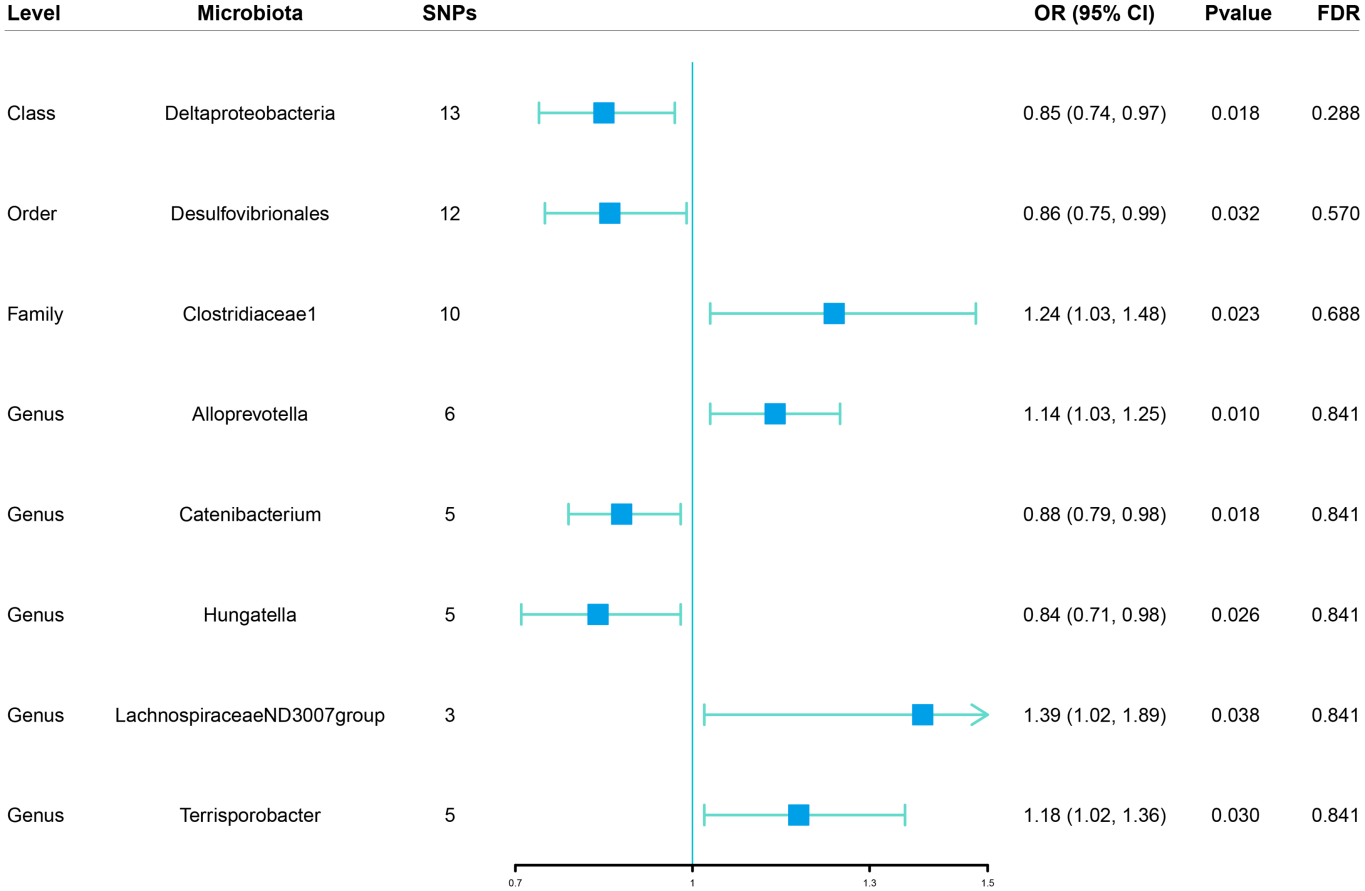
Associations of genetically predicted gut microbiota with sepsis risk using IVW method SNPs, single nucleotide polymorphisms; OR, odds ratio; CI, confidence interval; FDR, false discovery rate.

We implemented a two-sample MR to investigate the causal relationship between gut microbiome features and the risk of sepsis. If the gut microbiota feature contained only one IV, the Wald ratio test was applied in the MR analysis (Burgess et al., 2017). If the gut microbiota feature contained multiple IVs, the inverse variance weighted (IVW) test (Burgess et al., 2013) was adopted as the primary analysis approach, supplemented by other methods including MR-Egger, simple mode, weighted median, and weighted mode. In order to obtain an overall assessment of the impact of the gut microbiome on sepsis risk, the meta-analysis technique known as IVW turns the outcome effects of IVs on the exposure effects into a weighted regression with the intercept set to zero. In the absence of horizontal pleiotropy, IVW can provide estimates that are unbiased by avoiding the effects of confounders (Holmes et al., 2017). Additionally, the Benjamini and Hochberg false discovery rate (FDR) was adopted to correct our results for multiple hypothesis testing, with a significance level set at FDR-corrected value of *p* < 0.05 (Benjamini and Hochberg, 1995). Association that had a value of *p* < 0.05 but did not meet the FDR-controlled cutoff were considered suggestive (Figure 2). MR-Egger might be heavily impacted by outlier genetic variables, resulting in incorrect estimations. Although all of the chosen IVs are invalid, the MR-Egger approach could nevertheless produce unbiased estimations (Bowden et al., 2016b). Simple mode offers robustness for pleiotropy despite being less powerful than IVW (Milne et al., 2017). The weighted median method is capable of providing precise and reliable effect estimates if at least 50% of the data from valid instruments are available (Bowden et al., 2016a). For genetic variables that defy the pleiotropy hypothesis, the weighted mode method is adaptable (Hartwig et al., 2017).

### 2.4. Sensitivity analysis

To determine whether heterogeneity and pleiotropy within IVs could bias the MR results, a set of sensitivity analyses were conducted to verify the robustness of significant results. The Cochran’s Q test and funnel plots were utilized to measure the heterogeneity among the chosen genetic instruments. As mentioned above, the PhenoScanner database was explored to examine if any of the enrolled SNPs were significantly linked (*p* < 5 × 10^−8^) to other phenotypes that affected sepsis risk independently of the gut microbiota, and ultimately pleiotropic SNPs would be removed from the MR estimates. To access potential horizontal pleiotropic effects of included IVs, we further performed the MR Egger intercept and Mendelian randomization pleiotropy residual sum and outlier (MR-PRESSO) global test. Meanwhile, the leave-one-out sensitivity analysis was conducted to confirm the accuracy and robustness of the causal effect estimates and to ascertain MR estimates are not driven by strong influence SNPs. Furthermore, the MR Steiger directionality test (Hemani et al., 2017) was administered to infer causal direction. The identified causal link may be regarded as directionally credible if the variance explained by the IVs on the exposure is greater than the outcome. Power computations were performed depending on the site^3^ (Brion et al., 2013) (Supplementary Table S5). All statistical analyses in our work, including MR analyses and sensitivity analyses, were carried out by applying the R packages “TwoSampleMR” and “MRPRESSO” with the publicly available R software (version 4.2.1).

## 3. Results

Two-sample Mendelian randomization of gut microbiota on sepsis.

### 3.1. Locus-wide significance level

**Figure 3:**
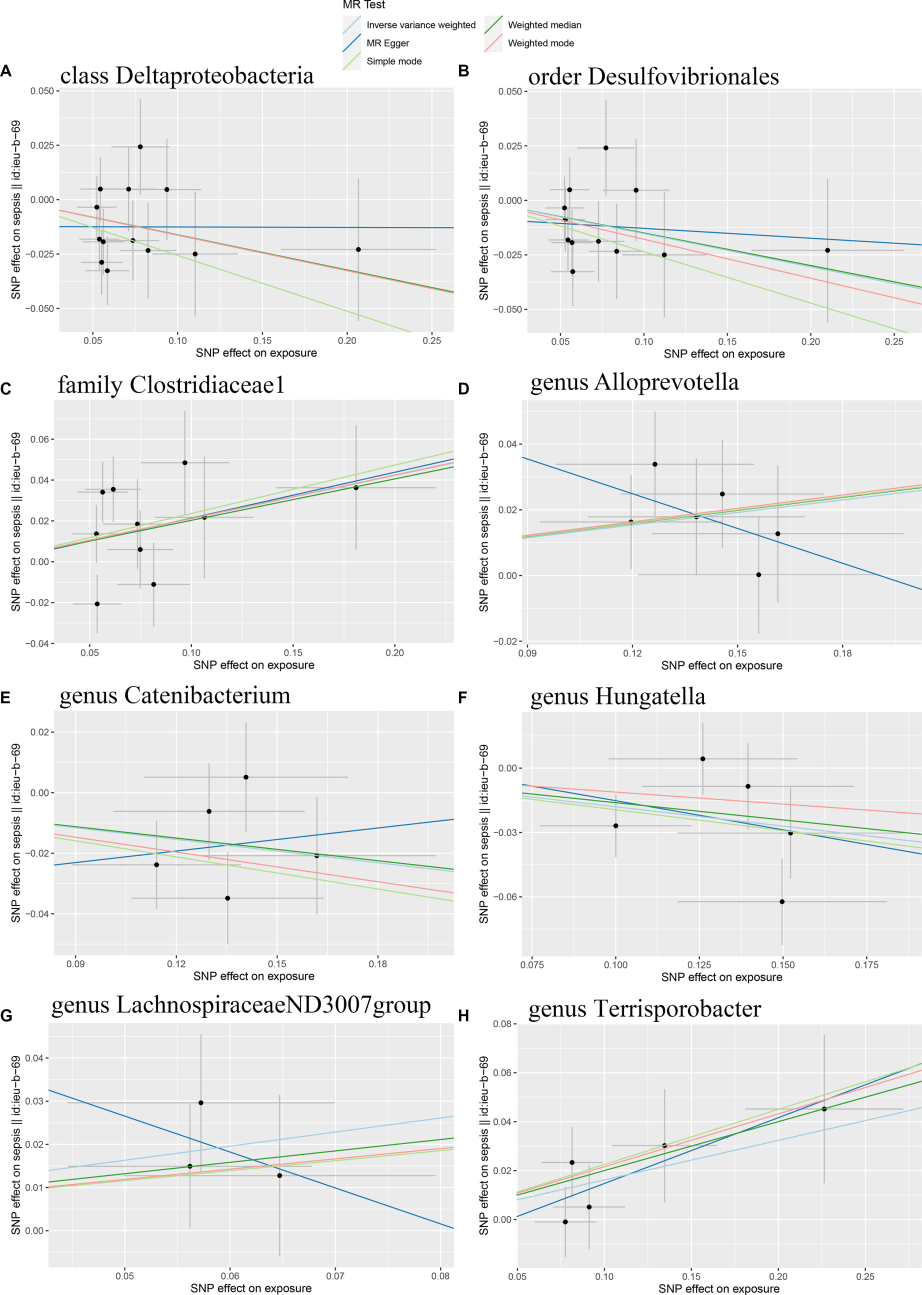
Scatter plots of the causal effect of gut microbiota on sepsis risk **(A)** class *Deltaproteobacteria*; **(B)** order *Desulfovibrionales*; **(C)** family *Clostridiaceae1*; **(D)** genus *Alloprevotella*; **(E)** genus *Catenibacterium*; **(F)** genus *Hungatella*; **(G)** genus *LachnospiraceaeND3007group*; **(H)** genus *Terrisporobacter*. The slope of the line represents the causality of the different MR methods.

Initially, 14,587 SNPs correlated with 211 gut microbiota traits (including 9 phyla, 16 classes, 20 orders, 35 families and 131 genera) at locus-wide significance level (*p* < 1 × 10^−5^) were determined as genetic instruments from large-scale GWAS generated by the MiBioGen consortium. Supplementary Table S2 provided detailed information on the selected SNPs, including effect allele, other allele, beta, SE, value of *p*, proxy SNP, etc. Following a series of screening criteria described above, MR analyses were done for each pair of exposure (bacteria taxa) and outcome (sepsis) to investigate causal relationship based on five MR methods (IVW, MR Egger, simple mode, weighted median, and weighted mode) (Supplementary Table S1). The results of reaching the threshold of *p* < 0.05 according to the IVW method are shown in Figure 2. The odds ratio (OR), which represented an elevated risk of sepsis per standard deviation increase in gut microbiota feature abundance, was used to quantify the causal effects. The results of IVW analyses demonstrated suggestive causal effects of the genetically predicted increased abundance of *Deltaproteobacteria* at the class level (OR, 0.85; 95% confidence interval [CI], 0.74–0.97; *p* = 0.018) and *Desulfovibrionales* at the order level (OR, 0.86; 95% CI, 0.75–0.99; *p* = 0.032) had protective effects on sepsis risk, while host-genetic-driven increased in *Clostridiaceae1* at the family level (OR, 1.24; 95% CI, 1.03–1.48; *p* = 0.023) were associated with higher risk of sepsis. We also found suggestive associations at the genus level that *Catenibacterium* (OR, 0.88; 95% CI, 0.79–0.98; *p* = 0.018) and *Hungatella* (OR, 0.84; 95% CI, 0.71–0.98; *p* = 0.026) were negatively linked to the risk of sepsis and *Alloprevotella* (OR, 1.14; 95% CI, 1.03–1.25; *p* = 0.010), *LachnospiraceaeND3007group* (OR, 1.39; 95% CI, 1.02–1.89; *p* = 0.038) and *Terrisporobacter* (OR, 1.18; 95% CI, 1.02–1.36; *p* = 0.030) were positively correlated with sepsis risk. The results of other complementary analytical methods were consistent in direction with the primary analysis, which reinforced confidence in the true causal association (Table 1). The scatter plot reflects the causal effects between gut microbiota and sepsis (Figure 3). However, all FDR-corrected *p*-values were greater than 0.05, indicating no significant associations (Figure 2). Detailed statistics for the 211 gut microbiota taxa were presented in Supplementary Table S1.

**Table 1:** MR estimates for the association between gut microbiota and sepsis (*p* < 1 × 10^−5^).

Level	Microbiota	SNPs	*R*^2^ (%)	Methods	Beta	OR (95% CI)	*p* value
Class	*Deltaproteobacteria*	13	6.0	Inverse variance weighted	−0.16	0.85 (0.74, 0.97)	0.018
				MR Egger	−0.00	1.00 (0.68, 1.46)	0.992
				Simple mode	−0.26	0.77 (0.56, 1.06)	0.139
				Weighted median	−0.16	0.85 (0.70, 1.03)	0.097
				Weighted mode	−0.16	0.85 (0.65, 1.12)	0.262
Order	*Desulfovibrionales*	12	5.6	Inverse variance weighted	−0.15	0.86 (0.75, 0.99)	0.032
				MR Egger	−0.05	0.96 (0.66, 1.38)	0.815
				Simple mode	−0.24	0.79 (0.58, 1.09)	0.174
				Weighted median	−0.15	0.86 (0.71, 1.04)	0.129
				Weighted mode	−0.18	0.84 (0.65, 1.08)	0.191
Family	*Clostridiaceae1*	10	4.5	Inverse variance weighted	0.21	1.24 (1.03, 1.48)	0.023
				MR Egger	0.22	1.25 (0.72, 2.17)	0.448
				Simple mode	0.24	1.27 (0.87, 1.85)	0.249
				Weighted median	0.20	1.22 (0.98, 1.54)	0.079
				Weighted mode	0.21	1.24 (0.91, 1.68)	0.210
Genus	*Alloprevotella*	6	6.5	Inverse variance weighted	0.13	1.14 (1.03, 1.25)	0.010
				MR Egger	−0.35	0.70 (0.28, 1.74)	0.488
				Simple mode	0.13	1.14 (0.95, 1.37)	0.211
				Weighted median	0.13	1.14 (1.01, 1.29)	0.036
				Weighted mode	0.14	1.15 (0.96, 1.36)	0.187
	*Catenibacterium*	5	5.2	Inverse variance weighted	−0.13	0.88 (0.79, 0.98)	0.018
				MR Egger	0.13	1.14 (0.40, 3.21)	0.826
				Simple mode	−0.18	0.84 (0.68, 1.04)	0.182
				Weighted median	−0.12	0.88 (0.76, 1.03)	0.110
				Weighted mode	−0.16	0.85 (0.68, 1.06)	0.217
	*Hungatella*	5	4.2	Inverse variance weighted	−0.18	0.84 (0.71, 0.98)	0.026
				MR Egger	−0.27	0.76 (0.24, 2.41)	0.675
				Simple mode	−0.19	0.82 (0.63, 1.08)	0.227
				Weighted median	−0.16	0.85 (0.71, 1.02)	0.081
				Weighted mode	−0.11	0.89 (0.69, 1.15)	0.440
	*LachnospiraceaeND3007group*	3	2.4	Inverse variance weighted	0.33	1.39 (1.02, 1.89)	0.038
				MR Egger	−0.83	0.44 (0.00, 78.11)	0.806
				Simple mode	0.23	1.26 (0.82, 1.93)	0.399
				Weighted median	0.26	1.30 (0.89, 1.91)	0.175
				Weighted mode	0.24	1.27 (0.81, 1.98)	0.404
	*Terrisporobacter*	5	3.5	Inverse variance weighted	0.16	1.18 (1.02, 1.36)	0.030
				MR Egger	0.27	1.31 (0.88, 1.95)	0.280
				Simple mode	0.23	1.25 (0.97, 1.61)	0.155
				Weighted median	0.20	1.22 (1.01, 1.47)	0.034
				Weighted mode	0.22	1.24 (0.97, 1.59)	0.163

SNPs, the number of SNPs being used as IVs; *R*^2^, the proportion of phenotypic variation explained by SNPs.

A total of eight causal effects were identified from gut microbiota taxa to sepsis. Of these, the F-statistic of the IVs ranged from 21.87 to 86.45, suggesting that there was no weak IV bias (Supplementary Table S4). The results of Cochran’s Q statistic for the IVW test showed no significant heterogeneity in these IVs (Table 2). Besides, the MR Egger intercept and MR-PRESSO global test were utilized to test for horizontal pleiotropy, and all *p*-values were greater than 0.05, indicating no significant directional horizontal pleiotropy (Table 2). No outliers were observed by MR-PRESSO analyses. In addition, the forest plots and the leave-one-out test revealed that no strong single SNP drives the MR estimation, illustrating the robustness of our findings (Supplementary Figures S1, S2). The MR Steiger directionality test demonstrated that the eight causal effects identified were robust in the direction from the gut microbiota to sepsis (Supplementary Table S4). Furthermore, our study had satisfactory power (more than 80%) to assess the causal effects of these gut microbiome features on sepsis (Supplementary Table S5). Of note, the order *Desulfovibrionales* is a subcategory of the class *Deltaproteobacteria*; therefore, the SNPs of these two sets may heavily overlap, as listed in Supplementary Table S4.

**Table 2:** Evaluation of heterogeneity and directional pleiotropy using different methods.

Level	Microbiota	Heterogeneity	Horizontal pleiotropy	
		Cochran’s Q *p*	MR-Egger intercept *p*	MR-PRESSO global test *p*
Class	*Deltaproteobacteria*	0.566	0.391	0.630
Order	*Desulfovibrionales*	0.708	0.555	0.758
Family	*Clostridiaceae1*	0.177	0.962	0.198
Genus	*Alloprevotella*	0.744	0.354	0.788
	*Catenibacterium*	0.437	0.661	0.480
	*Hungatella*	0.152	0.885	0.212
	*LachnospiraceaeND3007group*	0.694	0.737	/^*^
	*Terrisporobacter*	0.751	0.613	0.862

^*^Not enough instrumental variables for MR-PRESSO analysis.

### 3.2. Genome-wide statistical significance threshold

1,394 SNPs were identified as instrumental variables at genome-wide statistical significance threshold (*p* < 5 × 10^−8^). MR analyses were carried out following the removal of SNPs that had linkage disequilibrium effects, were strongly associated with confounders, and were palindromic. When the gut microbiota was considered as a whole, the IVW results (OR, 1.00; 95% CI, 0.92–1.09; *p* = 0.950) suggested no causal relationship between the gut microbiota and sepsis (Table 3). Details of the genetic variants were given in Supplementary Table S6. The results of Cochran’s Q test revealed no significant heterogeneity (*p* = 0.684). Additionally, no significant horizontal pleiotropy was found for the results of the MR-Egger intercept analysis (*p* = 0.823) and MR-PRESSO analysis (*p* = 0.712). The F-statistic of all selected SNPs was greater than 10. When gut microbiota was treated as individual bacterial abundance, MR analysis did not detect a causal association between gut microbiota features and the risk of sepsis (Table 3). However, as there were not enough IVs used in the MR to undertake a sensitivity analysis, the results should be interpreted with caution.

**Table 3:** MR estimates for the association between gut microbiota and sepsis (*p* < 5 × 10^−8^).

Level	Microbiota	SNPs	Methods	Beta	SE	OR (95% CI)	*p* value
Total		14	Inverse variance weighted	<0.01	0.04	1.00 (0.92, 1.09)	0.950
			MR Egger	0.04	0.15	1.04 (0.78, 1.38)	0.815
			Simple mode	−0.05	0.09	0.95 (0.80, 1.13)	0.581
			Weighted median	−0.02	0.06	0.98 (0.88, 1.10)	0.730
			Weighted mode	−0.02	0.07	0.98 (0.84, 1.13)	0.758
Phylum	*Actinobacteria*	1	Wald ratio	−0.26	0.20	0.77 (0.52, 1.15)	0.205
Class	*Actinobacteria*	1	Wald ratio	−0.20	0.15	0.82 (0.61, 1.11)	0.205
Order	*Bifidobacteriales*	2	Inverse variance weighted	−0.13	0.13	0.88 (0.68, 1.14)	0.322
	*Gastranaerophilales*	1	Wald ratio	0.15	0.14	1.17 (0.88, 1.54)	0.282
Family	*Bifidobacteriaceae*	2	Inverse variance weighted	−0.13	0.13	0.88 (0.68, 1.14)	0.322
	*Oxalobacteraceae*	1	Wald ratio	−0.08	0.14	0.92 (0.71, 1.20)	0.552
	*Peptostreptococcaceae*	1	Wald ratio	−0.14	0.21	0.87 (0.58, 1.30)	0.499
	*Streptococcaceae*	1	Wald ratio	0.22	0.22	1.24 (0.80, 1.93)	0.329
	*unknownfamily.id.1000001214*	1	Wald ratio	0.15	0.14	1.17 (0.88, 1.54)	0.282
Genus	*Allisonella*	1	Wald ratio	−0.02	0.10	0.98 (0.81, 1.18)	0.835
	*Bifidobacterium*	2	Inverse variance weighted	−0.23	0.15	0.80 (0.59, 1.07)	0.135
	*Enterorhabdus*	1	Wald ratio	0.21	0.15	1.23 (0.92, 1.64)	0.165
	*Erysipelatoclostridium*	1	Wald ratio	−0.06	0.17	0.94 (0.68, 1.32)	0.740
	*Eubacteriumcoprostanoligenesgroup*	1	Wald ratio	−0.25	0.23	0.78 (0.50, 1.22)	0.276
	*Oxalobacter*	1	Wald ratio	0.07	0.12	1.07 (0.84, 1.37)	0.561
	*Romboutsia*	1	Wald ratio	−0.14	0.20	0.87 (0.58, 1.30)	0.499
	*RuminococcaceaeUCG013*	1	Wald ratio	−0.21	0.23	0.81 (0.52, 1.27)	0.357
	*Ruminococcus1*	1	Wald ratio	0.24	0.22	1.27 (0.82, 1.97)	0.281
	*Streptococcus*	1	Wald ratio	0.21	0.21	1.23 (0.81, 1.87)	0.329
	*Tyzzerella3*	1	Wald ratio	−0.02	0.12	0.98 (0.78, 1.24)	0.867
	*unknowngenus.id.1000001215*	1	Wald ratio	0.15	0.14	1.17 (0.88, 1.54)	0.282

## 4. Discussion

Our MR study firstly assessed the potential causal link between gut microbiota and sepsis risk by leveraging large-scale summary statistics of microbiota GWAS and sepsis GWAS. A total of eight bacterial features (one at class level, one at order level, one at family level, and five at genus level) were identified to be suggestively causally correlated to the risk of sepsis. This study suggested that increased abundance of *Deltaproteobacteria*, *Desulfovibrionales*, *Catenibacterium*, and *Hungatella* were negatively associated with sepsis risk, while *Clostridiaceae1, Alloprevotella, LachnospiraceaeND3007group*, and *Terrisporobacter* may be risk factors for sepsis.

The gut has long been considered to be the motor of sepsis and multiple organ failure syndrome (Klingensmith and Coopersmith, 2015). Trillions of symbiotic gut microbiomes are densely populated on the gastrointestinal mucosal surface of the host, and their composition regulates the balance between host health and sickness. The loss of normal gut microbiota structure and function has been linked to a variety of disorders such as inflammatory bowel disease, *Clostridium difficile* infection, and obesity (Shreiner et al., 2015). There is growing evidence that disturbance of the gut microbiome predisposes to sepsis and has a detrimental influence on sepsis outcomes, even though the pathophysiology of sepsis is multifaceted and insufficiently understood. Studies have shown that the composition of the gut microbiota of ICU patients differs dramatically from that of healthy controls (Zaborin et al., 2014; Ojima et al., 2016). Several pathogenic and antibiotic-resistant microorganisms, such as the genera *Staphylococcus* and *Enterococcus*, overload the gut during sepsis, while the gut loses essential bacterial genera that are a significant component of the microbiota in healthy individuals. Examples include *Prevotella*, *Blautia*, and *Ruminococcaceae*, which are known to generate short-chain fatty acids (SCFAs) (Zaborin et al., 2014; Ojima et al., 2016). SCFAs strengthen the barrier function of the intestinal epithelium and exert anti-inflammatory effects (Kamada et al., 2013) on epithelial cells that are partly mediated through histone deacetylase (Li et al., 2018).To protect the host against pathogen colonization, the gut microbiota, immune system, and gut epithelial barrier are all tightly interlinked (Kamada et al., 2013). The specific gut microbiota changes during sepsis are a crucial event in critical care research (Schuijt et al., 2013). The gut has been implicated to play a significant role in the systemic inflammatory response syndrome (SIRS) found in critically ill patients (Taylor, 1998). Due to mucosal hypoxia or stress, the intestinal barrier is disrupted and the permeability of the epithelial barrier is compromised, leading to enhanced bacterial translocation and a role in multi-organ failure and sepsis (Taylor, 1998). The composition of the gut microbiota, as well as the status of the mucosal barriers, are altered during critical illness. In critically ill patients, changes in the composition of the microbiota, such as a reduction in total bacterial count and diversity, can lead to systemic dysregulation (Schuijt et al., 2013). The ratios of *Bacteroidetes* to *Firmicutes* in the ICU altered dramatically over time, and it may be employed as a prognostic factor in critically ill patients in the future (Ojima et al., 2016). In addition, studies have shown that changes in the composition of the gut microbiota are associated with morbidity and mortality in patients with SIRS (Shimizu et al., 2006, 2011). Modulating the microbiota composition in the gut by supporting the microbiota or treating patients with selected microbial products may be a promising therapeutic strategy for critically ill or septic patients.

Our work identified eight specific bacterial features with causal effects on the risk of sepsis. Among them, Class *Deltaproteobacteria* and order *Desulfovibrionales* belong to phylum *Proteobacteria*, while family *Clostridiaceae1*, genus *Catenibacterium, Hungatella, LachnospiraceaeND3007group,* and *Terrisporobacter* belong to phylum *Firmicutes*. A prospective, observational case–control study showed that the percentage of *Firmicutes* at the phylum level was considerably lower in septic patients than in healthy controls, the proportions of *Proteobacteria* and *Bacteroidetes*, including lipopolysaccharide-containing bacteria and pathogenic like *Klebsiella* and *Escherichia*, were considerably higher in septic patients than in healthy controls (Mu et al., 2022). However, such specific intestinal flora variations varied in different studies. The results of an experimental animal study showed that the cecal ligation and puncture (CLP) group had a significantly increased *Firmicutes/Bacteroidetes* ratio and a decreased relative abundance of *Escherichia* and *Alloprevotella* compared to the control group rats (Zhao et al., 2022). It is consistent with another animal model analysis, where the abundance of *Alloprevotella* was significantly lower in CLP groups compared to the sham-operated group (Li et al., 2022). The gram-negative bacillus, *Alloprevotella*, is related to short-chain fatty acid production and anti-inflammation and is often considered a probiotic. However, we found that *Alloprevotella* at the genus level is a risk factor for sepsis, which is contrary to these studies. Though *Alloprevotella* is typically thought of as a helpful bacterium, different species and strains may affect sepsis in various ways.

A previous study revealed that the abundance of *Catenibacterium*, an intestinal probiotic, decreased in patients with sepsis-associated encephalopathy (Wang et al., 2022). Consistent with this study, our findings suggested that the abundance of genus *Catenibacterium* was negatively linked to sepsis risk. The *Lachnospiraceae* family, which is abundant and exclusively anaerobic in the healthy gut, has an impact on its hosts by transforming primary bile acids into secondary bile acids, producing short-chain fatty acids, and resisting colonization by intestinal pathogens. *Lachnospiraceae* were linked to protective effects on metabolic health in sepsis (Sorbara et al., 2020). However, our results suggested that the genetically predicted abundance of *LachnospiraceaeND3007group* at the genus level was positively correlated with sepsis risk. Our findings were supported by a study’s discovery that the family *Lachnospiraceae* increased in the subacute phase (day 7 after CLP) in a murine sepsis model (Muratsu et al., 2022). Inconsistent results remind us that there is considerable inter- and intra-species diversity affecting host health, and that a standardized and more specific gut microbiota classification system is essential for subsequent mechanistic studies and clinical guidance. We found suggestive evidence of causal effects of class *Deltaproteobacteria*, order *Desulfovibrionales*, family *Clostridiaceae1*, and genus *Hungatella* and *Terrisporobacter* with sepsis, however, no relevant result was presented in earlier research. Decades of studies on sepsis have made it clear that the pathway and outcome of sepsis are greatly impacted by host genetics and environmental factors (Lee and Banerjee, 2020). To improve primary prevention and assist the development of mechanism-guided treatment, a deeper understanding of the host-genetic-driven gut microbiota associated with sepsis is critically required. Although our MR steiger results indicated causality from gut microbiota to sepsis, it could not be excluded that sepsis also affects gut flora ecology; the interplay between gut microbiota and sepsis needs further studies.

Our study has multiple strengths. To start with, this is the first study to explore the causal effect of gut microbiota on the risk of sepsis by MR analysis. Results from MR analysis may be more credible than those from conventional observational research because it lessens the bias caused by confounders and reverse causality. The identified causal associations may present candidate intestinal bacteria for follow-up mechanistic investigations. Secondly, SNPs associated with gut microbiota were taken from the largest GWAS meta-analysis up to date, guaranteeing the strength of IVs in our study. In addition, the enhanced statistical power of the large sample size datasets and the application of multiple sensitivity analyses ensure the robustness of our findings. Nevertheless, certain limitations warrant consideration. First, although the overwhelming majority of individuals in the gut microbiota GWAS utilized in our MR analysis were of European descent, a small amount of data were obtained from non-European descent, which may partially bias our results. In addition, extrapolation of our findings to other races may be restricted. Second, our findings did not meet the strict FDR correction. However, MR was a hypothesis-driven method that can be used to test some causal effects without FDR adjustment when there exists some biological plausibility. Third, we were unable to undertake subgroup analyses, such as differentiating between early-onset and late-onset sepsis, because our study used summary-level data on sepsis rather than raw data. Moreover, the non-linear relationship between gut microbiota and sepsis cannot be examined on the basis of standard MR. Finally, there is a lack of direct mechanistic studies to support our results. To elucidate the relationship between gut microbiota and sepsis, more efforts are needed to investigate the effects of gut flora on the immune system, intestinal barrier, pathogens, and disease susceptibility pathways.

## 5. Conclusion

In summary, our study suggests genetic evidence of causal effects of gut microbiota on sepsis. The beneficial or detrimental gut microbiota identified in this study for the risk of sepsis may provide valuable insights into the pathogenesis of microbiota-mediated sepsis and strategies for sepsis prevention and treatment.

## Data availability statement

The original contributions presented in the study are included in the article/Supplementary material, further inquiries can be directed to the corresponding authors.

## Ethics statement

Ethical review and approval was not required for the study on human participants in accordance with the local legislation and institutional requirements. Written informed consent for participation was not required for this study in accordance with the national legislation and the institutional requirements.

## Author contributions

J-HC, L-YZ, and Y-FZ: study conception, design, data analyses, and draft preparation. H-XT, HL, and Y-FW: literature search. K-XL and Y-QD: supervision of the study. All authors contributed to the article and approved the submitted version.

## Funding

This work was supported by grants from the National Natural Science Foundation of China (82172141).

## Conflict of interest

The authors declare that the research was conducted in the absence of any commercial or financial relationships that could be construed as a potential conflict of interest.

## Publisher’s note

All claims expressed in this article are solely those of the authors and do not necessarily represent those of their affiliated organizations, or those of the publisher, the editors and the reviewers. Any product that may be evaluated in this article, or claim that may be made by its manufacturer, is not guaranteed or endorsed by the publisher.

## Footnotes

^1^ https://snipa.helmholtz-muenchen.de/snipa3/

^2^ http://www.phenoscanner.medschl.cam.ac.uk/

^3^ https://shiny.cnsgenomics.com/mRnd/

## Abbreviations

MR: Mendelian randomization
GWAS: Genome-wide association study
IV: Instrumental variable
SNP: Single nucleotide polymorphism
IVW: Inverse variance weighted
MR-PRESSO: MR polymorphism residual sum and outlier
ICU: Intensive care unit
FDR: False discovery rate
OR: Odds ratio
CI: Confidence interval
SCFAs: Short-chain fatty acids
SIRS: Systemic inflammatory response syndrome
CLP: Cecal ligation and puncture
