# Supplementary material for: Causal effects of gut microbiota on sepsis: a two-sample Mendelian randomization study
Source: Front Microbiol. 2023 May 10;14:1167416. doi: 10.3389/fmicb.2023.1167416 (PMC10206031; doi:10.3389/fmicb.2023.1167416)
Supplement: Supplementary file 1 [file Table_1.docx]

**Supplementary Table 1. The estimates of two-sample MR of gut microbiome and sepsis risk**

| **Feature** | **Taxa** | **SNPs** | **Methods** | **Beta** | **SE** | **OR (95% CI)** | **P value** |
| --- | --- | --- | --- | --- | --- | --- | --- |
| Phylum | Actinobacteria.id.400 | 15 | Inverse variance weighted | -0.06 | 0.09 | 0.94 (0.79, 1.11) | 0.492 |
|  |  |  | MR Egger | -0.55 | 0.33 | 0.58 (0.30, 1.10) | 0.117 |
|  |  |  | Simple mode | -0.11 | 0.19 | 0.90 (0.62, 1.30) | 0.577 |
|  |  |  | Weighted median | -0.08 | 0.10 | 0.92 (0.76, 1.12) | 0.420 |
|  |  |  | Weighted mode | -0.11 | 0.18 | 0.90 (0.63, 1.29) | 0.578 |
|  | Bacteroidetes.id.905 | 12 | Inverse variance weighted | 0.05 | 0.07 | 1.06 (0.91, 1.22) | 0.474 |
|  |  |  | MR Egger | 0.06 | 0.16 | 1.06 (0.78, 1.45) | 0.698 |
|  |  |  | Simple mode | 0.10 | 0.16 | 1.10 (0.81, 1.50) | 0.537 |
|  |  |  | Weighted median | 0.09 | 0.10 | 1.09 (0.89, 1.33) | 0.401 |
|  |  |  | Weighted mode | 0.15 | 0.15 | 1.16 (0.87, 1.54) | 0.335 |
|  | Cyanobacteria.id.1500 | 8 | Inverse variance weighted | 0.05 | 0.07 | 1.05 (0.92, 1.20) | 0.490 |
|  |  |  | MR Egger | 0.32 | 0.22 | 1.37 (0.89, 2.14) | 0.206 |
|  |  |  | Simple mode | -0.08 | 0.14 | 0.92 (0.70, 1.22) | 0.588 |
|  |  |  | Weighted median | 0.04 | 0.08 | 1.05 (0.90, 1.22) | 0.565 |
|  |  |  | Weighted mode | 0.10 | 0.14 | 1.11 (0.85, 1.44) | 0.483 |
|  | Euryarchaeota.id.55 | 12 | Inverse variance weighted | 0.05 | 0.04 | 1.05 (0.97, 1.14) | 0.239 |
|  |  |  | MR Egger | 0.29 | 0.18 | 1.33 (0.94, 1.90) | 0.137 |
|  |  |  | Simple mode | 0.02 | 0.10 | 1.02 (0.84, 1.24) | 0.847 |
|  |  |  | Weighted median | 0.03 | 0.06 | 1.03 (0.92, 1.15) | 0.605 |
|  |  |  | Weighted mode | 0.02 | 0.10 | 1.02 (0.84, 1.24) | 0.835 |
|  | Firmicutes.id.1672 | 16 | Inverse variance weighted | 0.10 | 0.07 | 1.11 (0.97, 1.27) | 0.143 |
|  |  |  | MR Egger | 0.05 | 0.18 | 1.05 (0.73, 1.51) | 0.795 |
|  |  |  | Simple mode | 0.25 | 0.18 | 1.28 (0.91, 1.82) | 0.181 |
|  |  |  | Weighted median | 0.11 | 0.10 | 1.11 (0.92, 1.34) | 0.279 |
|  |  |  | Weighted mode | 0.14 | 0.15 | 1.15 (0.85, 1.55) | 0.378 |
|  | Lentisphaerae.id.2238 | 9 | Inverse variance weighted | -0.05 | 0.05 | 0.95 (0.87, 1.05) | 0.334 |
|  |  |  | MR Egger | -0.21 | 0.18 | 0.81 (0.57, 1.14) | 0.262 |
|  |  |  | Simple mode | -0.03 | 0.10 | 0.97 (0.81, 1.17) | 0.794 |
|  |  |  | Weighted median | -0.03 | 0.06 | 0.97 (0.87, 1.09) | 0.612 |
|  |  |  | Weighted mode | -0.02 | 0.09 | 0.98 (0.82, 1.16) | 0.791 |
|  | Proteobacteria.id.2375 | 12 | Inverse variance weighted | -0.11 | 0.12 | 0.90 (0.72, 1.13) | 0.362 |
|  |  |  | MR Egger | 0.45 | 0.32 | 1.57 (0.83, 2.95) | 0.193 |
|  |  |  | Simple mode | 0.17 | 0.21 | 1.18 (0.79, 1.77) | 0.438 |
|  |  |  | Weighted median | 0.10 | 0.12 | 1.10 (0.87, 1.40) | 0.422 |
|  |  |  | Weighted mode | 0.16 | 0.18 | 1.18 (0.82, 1.68) | 0.394 |
|  | Tenericutes.id.3919 | 12 | Inverse variance weighted | -0.01 | 0.07 | 0.99 (0.87, 1.13) | 0.897 |
|  |  |  | MR Egger | 0.04 | 0.23 | 1.04 (0.66, 1.64) | 0.855 |
|  |  |  | Simple mode | 0.10 | 0.13 | 1.10 (0.85, 1.43) | 0.486 |
|  |  |  | Weighted median | -0.00 | 0.10 | 1.00 (0.84, 1.19) | 0.982 |
|  |  |  | Weighted mode | 0.07 | 0.12 | 1.07 (0.84, 1.36) | 0.607 |
|  | Verrucomicrobia.id.3982 | 12 | Inverse variance weighted | 0.07 | 0.07 | 1.07 (0.94, 1.21) | 0.315 |
|  |  |  | MR Egger | 0.00 | 0.19 | 1.00 (0.69, 1.45) | 0.988 |
|  |  |  | Simple mode | 0.09 | 0.13 | 1.10 (0.85, 1.41) | 0.494 |
|  |  |  | Weighted median | 0.06 | 0.09 | 1.06 (0.89, 1.26) | 0.485 |
|  |  |  | Weighted mode | 0.08 | 0.13 | 1.08 (0.84, 1.39) | 0.560 |
| Class | Actinobacteria.id.419 | 15 | Inverse variance weighted | -0.12 | 0.07 | 0.89 (0.77, 1.02) | 0.091 |
|  |  |  | MR Egger | -0.04 | 0.21 | 0.96 (0.63, 1.46) | 0.847 |
|  |  |  | Simple mode | -0.09 | 0.16 | 0.91 (0.67, 1.24) | 0.573 |
|  |  |  | Weighted median | -0.12 | 0.09 | 0.89 (0.74, 1.07) | 0.208 |
|  |  |  | Weighted mode | -0.12 | 0.15 | 0.89 (0.67, 1.18) | 0.427 |
|  | Alphaproteobacteria.id.2379 | 7 | Inverse variance weighted | 0.00 | 0.08 | 1.00 (0.86, 1.17) | 0.956 |
|  |  |  | MR Egger | -0.14 | 0.30 | 0.87 (0.48, 1.57) | 0.653 |
|  |  |  | Simple mode | -0.04 | 0.15 | 0.96 (0.72, 1.28) | 0.801 |
|  |  |  | Weighted median | -0.03 | 0.10 | 0.97 (0.79, 1.20) | 0.805 |
|  |  |  | Weighted mode | -0.03 | 0.15 | 0.97 (0.72, 1.30) | 0.832 |
|  | Bacilli.id.1673 | 17 | Inverse variance weighted | 0.05 | 0.06 | 1.05 (0.92, 1.19) | 0.463 |
|  |  |  | MR Egger | 0.07 | 0.17 | 1.08 (0.77, 1.51) | 0.667 |
|  |  |  | Simple mode | 0.17 | 0.15 | 1.18 (0.87, 1.59) | 0.296 |
|  |  |  | Weighted median | 0.06 | 0.09 | 1.06 (0.89, 1.27) | 0.491 |
|  |  |  | Weighted mode | 0.09 | 0.13 | 1.09 (0.84, 1.42) | 0.508 |
|  | Bacteroidia.id.912 | 14 | Inverse variance weighted | 0.03 | 0.07 | 1.03 (0.90, 1.19) | 0.654 |
|  |  |  | MR Egger | 0.15 | 0.15 | 1.17 (0.86, 1.57) | 0.334 |
|  |  |  | Simple mode | 0.12 | 0.17 | 1.13 (0.82, 1.57) | 0.471 |
|  |  |  | Weighted median | 0.08 | 0.10 | 1.08 (0.89, 1.32) | 0.424 |
|  |  |  | Weighted mode | 0.15 | 0.14 | 1.16 (0.89, 1.52) | 0.298 |
|  | Betaproteobacteria.id.2867 | 12 | Inverse variance weighted | -0.09 | 0.09 | 0.92 (0.76, 1.10) | 0.343 |
|  |  |  | MR Egger | 0.13 | 0.33 | 1.14 (0.59, 2.20) | 0.704 |
|  |  |  | Simple mode | 0.02 | 0.22 | 1.02 (0.67, 1.56) | 0.932 |
|  |  |  | Weighted median | 0.01 | 0.12 | 1.01 (0.80, 1.26) | 0.964 |
|  |  |  | Weighted mode | 0.04 | 0.18 | 1.04 (0.73, 1.49) | 0.821 |
|  | Clostridia.id.1859 | 12 | Inverse variance weighted | 0.04 | 0.09 | 1.04 (0.88, 1.23) | 0.657 |
|  |  |  | MR Egger | 0.31 | 0.45 | 1.37 (0.57, 3.29) | 0.500 |
|  |  |  | Simple mode | -0.12 | 0.19 | 0.89 (0.61, 1.29) | 0.540 |
|  |  |  | Weighted median | 0.00 | 0.11 | 1.00 (0.80, 1.25) | 0.977 |
|  |  |  | Weighted mode | -0.11 | 0.20 | 0.90 (0.61, 1.32) | 0.587 |
|  | Coriobacteriia.id.809 | 13 | Inverse variance weighted | -0.06 | 0.08 | 0.94 (0.80, 1.10) | 0.441 |
|  |  |  | MR Egger | 0.17 | 0.32 | 1.19 (0.63, 2.22) | 0.601 |
|  |  |  | Simple mode | -0.14 | 0.16 | 0.87 (0.63, 1.20) | 0.414 |
|  |  |  | Weighted median | -0.08 | 0.11 | 0.92 (0.75, 1.13) | 0.428 |
|  |  |  | Weighted mode | -0.13 | 0.16 | 0.88 (0.64, 1.21) | 0.454 |
|  | Deltaproteobacteria.id.3087 | 13 | Inverse variance weighted | -0.16 | 0.07 | 0.85 (0.74, 0.97) | 0.018 |
|  |  |  | MR Egger | -0.00 | 0.19 | 1.00 (0.68, 1.46) | 0.992 |
|  |  |  | Simple mode | -0.26 | 0.16 | 0.77 (0.56, 1.06) | 0.139 |
|  |  |  | Weighted median | -0.16 | 0.10 | 0.85 (0.70, 1.03) | 0.097 |
|  |  |  | Weighted mode | -0.16 | 0.14 | 0.85 (0.65, 1.12) | 0.262 |
|  | Erysipelotrichia.id.2147 | 13 | Inverse variance weighted | 0.04 | 0.09 | 1.04 (0.88, 1.23) | 0.642 |
|  |  |  | MR Egger | -0.04 | 0.38 | 0.96 (0.46, 2.02) | 0.920 |
|  |  |  | Simple mode | -0.06 | 0.20 | 0.94 (0.63, 1.40) | 0.774 |
|  |  |  | Weighted median | 0.03 | 0.11 | 1.03 (0.83, 1.28) | 0.803 |
|  |  |  | Weighted mode | -0.03 | 0.19 | 0.97 (0.67, 1.39) | 0.858 |
|  | Gammaproteobacteria.id.3303 | 7 | Inverse variance weighted | 0.09 | 0.10 | 1.09 (0.90, 1.32) | 0.389 |
|  |  |  | MR Egger | 0.13 | 0.33 | 1.13 (0.59, 2.16) | 0.718 |
|  |  |  | Simple mode | -0.10 | 0.19 | 0.91 (0.62, 1.32) | 0.636 |
|  |  |  | Weighted median | 0.02 | 0.13 | 1.02 (0.80, 1.31) | 0.869 |
|  |  |  | Weighted mode | -0.09 | 0.21 | 0.91 (0.61, 1.37) | 0.677 |
|  | Lentisphaeria.id.2250 | 8 | Inverse variance weighted | -0.05 | 0.05 | 0.95 (0.86, 1.05) | 0.340 |
|  |  |  | MR Egger | -0.21 | 0.17 | 0.81 (0.58, 1.14) | 0.272 |
|  |  |  | Simple mode | -0.02 | 0.09 | 0.98 (0.81, 1.18) | 0.804 |
|  |  |  | Weighted median | -0.04 | 0.06 | 0.96 (0.85, 1.09) | 0.563 |
|  |  |  | Weighted mode | -0.02 | 0.09 | 0.98 (0.82, 1.17) | 0.805 |
|  | Melainabacteria.id.1589 | 10 | Inverse variance weighted | -0.03 | 0.07 | 0.97 (0.85, 1.11) | 0.655 |
|  |  |  | MR Egger | 0.26 | 0.18 | 1.30 (0.92, 1.84) | 0.171 |
|  |  |  | Simple mode | -0.00 | 0.16 | 1.00 (0.73, 1.35) | 0.978 |
|  |  |  | Weighted median | 0.00 | 0.08 | 1.00 (0.85, 1.18) | 0.990 |
|  |  |  | Weighted mode | 0.06 | 0.14 | 1.06 (0.81, 1.39) | 0.668 |
|  | Methanobacteria.id.119 | 10 | Inverse variance weighted | 0.03 | 0.05 | 1.03 (0.94, 1.13) | 0.522 |
|  |  |  | MR Egger | 0.16 | 0.20 | 1.17 (0.79, 1.74) | 0.451 |
|  |  |  | Simple mode | 0.06 | 0.09 | 1.06 (0.88, 1.27) | 0.566 |
|  |  |  | Weighted median | 0.05 | 0.06 | 1.05 (0.94, 1.17) | 0.359 |
|  |  |  | Weighted mode | 0.06 | 0.10 | 1.06 (0.87, 1.28) | 0.578 |
|  | Mollicutes.id.3920 | 12 | Inverse variance weighted | -0.01 | 0.07 | 0.99 (0.87, 1.13) | 0.897 |
|  |  |  | MR Egger | 0.04 | 0.23 | 1.04 (0.66, 1.64) | 0.855 |
|  |  |  | Simple mode | 0.10 | 0.14 | 1.10 (0.83, 1.46) | 0.522 |
|  |  |  | Weighted median | -0.00 | 0.10 | 1.00 (0.83, 1.20) | 0.982 |
|  |  |  | Weighted mode | 0.07 | 0.11 | 1.07 (0.85, 1.33) | 0.577 |
|  | Negativicutes.id.2164 | 12 | Inverse variance weighted | 0.04 | 0.12 | 1.04 (0.83, 1.31) | 0.729 |
|  |  |  | MR Egger | 0.13 | 0.39 | 1.14 (0.53, 2.43) | 0.746 |
|  |  |  | Simple mode | 0.13 | 0.24 | 1.13 (0.71, 1.82) | 0.613 |
|  |  |  | Weighted median | -0.03 | 0.12 | 0.97 (0.76, 1.22) | 0.775 |
|  |  |  | Weighted mode | 0.03 | 0.20 | 1.03 (0.69, 1.52) | 0.899 |
|  | Verrucomicrobiae.id.4029 | 11 | Inverse variance weighted | 0.03 | 0.07 | 1.03 (0.90, 1.17) | 0.680 |
|  |  |  | MR Egger | -0.04 | 0.25 | 0.96 (0.59, 1.56) | 0.875 |
|  |  |  | Simple mode | 0.08 | 0.13 | 1.08 (0.85, 1.39) | 0.544 |
|  |  |  | Weighted median | 0.05 | 0.09 | 1.06 (0.89, 1.25) | 0.528 |
|  |  |  | Weighted mode | 0.07 | 0.13 | 1.07 (0.83, 1.38) | 0.609 |
| Order | Actinomycetales.id.420 | 5 | Inverse variance weighted | 0.08 | 0.08 | 1.08 (0.93, 1.26) | 0.327 |
|  |  |  | MR Egger | -0.12 | 0.20 | 0.89 (0.60, 1.31) | 0.592 |
|  |  |  | Simple mode | -0.02 | 0.15 | 0.98 (0.72, 1.32) | 0.898 |
|  |  |  | Weighted median | 0.01 | 0.10 | 1.01 (0.84, 1.23) | 0.905 |
|  |  |  | Weighted mode | -0.03 | 0.12 | 0.97 (0.76, 1.24) | 0.835 |
|  | Bacillales.id.1674 | 9 | Inverse variance weighted | 0.05 | 0.05 | 1.05 (0.95, 1.17) | 0.314 |
|  |  |  | MR Egger | 0.29 | 0.25 | 1.34 (0.82, 2.17) | 0.279 |
|  |  |  | Simple mode | -0.07 | 0.12 | 0.93 (0.73, 1.18) | 0.557 |
|  |  |  | Weighted median | 0.00 | 0.06 | 1.00 (0.89, 1.13) | 0.995 |
|  |  |  | Weighted mode | -0.07 | 0.11 | 0.93 (0.75, 1.16) | 0.545 |
|  | Bacteroidales.id.913 | 14 | Inverse variance weighted | 0.03 | 0.07 | 1.03 (0.90, 1.19) | 0.654 |
|  |  |  | MR Egger | 0.15 | 0.15 | 1.17 (0.86, 1.57) | 0.334 |
|  |  |  | Simple mode | 0.12 | 0.16 | 1.13 (0.82, 1.56) | 0.463 |
|  |  |  | Weighted median | 0.08 | 0.10 | 1.08 (0.89, 1.32) | 0.421 |
|  |  |  | Weighted mode | 0.15 | 0.14 | 1.16 (0.89, 1.52) | 0.298 |
|  | Bifidobacteriales.id.432 | 11 | Inverse variance weighted | -0.05 | 0.09 | 0.95 (0.82, 1.13) | 0.551 |
|  |  |  | MR Egger | -0.01 | 0.42 | 0.99 (0.43, 2.26) | 0.977 |
|  |  |  | Simple mode | -0.24 | 0.20 | 0.78 (0.53, 1.15) | 0.245 |
|  |  |  | Weighted median | -0.17 | 0.12 | 0.85 (0.67, 1.06) | 0.152 |
|  |  |  | Weighted mode | -0.24 | 0.19 | 0.78 (0.54, 1.14) | 0.237 |
|  | Burkholderiales.id.2874 | 11 | Inverse variance weighted | -0.01 | 0.08 | 0.99 (0.84, 1.17) | 0.926 |
|  |  |  | MR Egger | 0.03 | 0.28 | 1.03 (0.60, 1.77) | 0.917 |
|  |  |  | Simple mode | -0.09 | 0.20 | 0.91 (0.62, 1.34) | 0.644 |
|  |  |  | Weighted median | 0.01 | 0.12 | 1.01 (0.81, 1.27) | 0.920 |
|  |  |  | Weighted mode | -0.05 | 0.20 | 0.95 (0.64, 1.41) | 0.806 |
|  | Clostridiales.id.1863 | 13 | Inverse variance weighted | 0.06 | 0.08 | 1.07 (0.91, 1.25) | 0.431 |
|  |  |  | MR Egger | 0.39 | 0.41 | 1.48 (0.66, 3.31) | 0.357 |
|  |  |  | Simple mode | -0.09 | 0.19 | 0.92 (0.64, 1.32) | 0.647 |
|  |  |  | Weighted median | 0.02 | 0.11 | 1.02 (0.82, 1.27) | 0.864 |
|  |  |  | Weighted mode | -0.08 | 0.18 | 0.92 (0.64, 1.31) | 0.650 |
|  | Coriobacteriales.id.810 | 13 | Inverse variance weighted | -0.06 | 0.08 | 0.94 (0.80, 1.10) | 0.441 |
|  |  |  | MR Egger | 0.17 | 0.32 | 1.19 (0.63, 2.22) | 0.601 |
|  |  |  | Simple mode | -0.14 | 0.17 | 0.87 (0.62, 1.22) | 0.437 |
|  |  |  | Weighted median | -0.08 | 0.10 | 0.92 (0.75, 1.13) | 0.423 |
|  |  |  | Weighted mode | -0.13 | 0.17 | 0.88 (0.64, 1.22) | 0.460 |
|  | Desulfovibrionales.id.3156 | 12 | Inverse variance weighted | -0.15 | 0.07 | 0.86 (0.75, 0.99) | 0.032 |
|  |  |  | MR Egger | -0.05 | 0.19 | 0.96 (0.66, 1.38) | 0.815 |
|  |  |  | Simple mode | -0.24 | 0.16 | 0.79 (0.58, 1.09) | 0.174 |
|  |  |  | Weighted median | -0.15 | 0.10 | 0.86 (0.71, 1.04) | 0.129 |
|  |  |  | Weighted mode | -0.18 | 0.13 | 0.84 (0.65, 1.08) | 0.191 |
|  | Enterobacteriales.id.3468 | 6 | Inverse variance weighted | 0.20 | 0.11 | 1.22 (0.99, 1.51) | 0.057 |
|  |  |  | MR Egger | -0.25 | 0.63 | 0.78 (0.23, 2.67) | 0.710 |
|  |  |  | Simple mode | 0.23 | 0.22 | 1.26 (0.81, 1.95) | 0.352 |
|  |  |  | Weighted median | 0.23 | 0.14 | 1.25 (0.96, 1.63) | 0.098 |
|  |  |  | Weighted mode | 0.23 | 0.21 | 1.25 (0.84, 1.88) | 0.322 |
|  | Erysipelotrichales.id.2148 | 13 | Inverse variance weighted | 0.04 | 0.09 | 1.04 (0.88, 1.23) | 0.642 |
|  |  |  | MR Egger | -0.04 | 0.38 | 0.96 (0.46, 2.02) | 0.920 |
|  |  |  | Simple mode | -0.06 | 0.21 | 0.94 (0.63, 1.41) | 0.777 |
|  |  |  | Weighted median | 0.03 | 0.12 | 1.03 (0.82, 1.29) | 0.810 |
|  |  |  | Weighted mode | -0.03 | 0.19 | 0.97 (0.66, 1.42) | 0.864 |
|  | Gastranaerophilales.id.1591 | 9 | Inverse variance weighted | -0.03 | 0.08 | 0.97 (0.83, 1.14) | 0.739 |
|  |  |  | MR Egger | 0.27 | 0.20 | 1.31 (0.88, 1.96) | 0.221 |
|  |  |  | Simple mode | -0.23 | 0.17 | 0.80 (0.57, 1.12) | 0.226 |
|  |  |  | Weighted median | 0.01 | 0.08 | 1.01 (0.86, 1.18) | 0.927 |
|  |  |  | Weighted mode | 0.09 | 0.14 | 1.10 (0.83, 1.45) | 0.540 |
|  | Lactobacillales.id.1800 | 13 | Inverse variance weighted | 0.04 | 0.07 | 1.04 (0.91, 1.20) | 0.570 |
|  |  |  | MR Egger | 0.13 | 0.17 | 1.13 (0.81, 1.59) | 0.485 |
|  |  |  | Simple mode | 0.10 | 0.17 | 1.10 (0.78, 1.55) | 0.586 |
|  |  |  | Weighted median | 0.01 | 0.10 | 1.01 (0.83, 1.23) | 0.926 |
|  |  |  | Weighted mode | 0.02 | 0.14 | 1.02 (0.77, 1.35) | 0.882 |
|  | Methanobacteriales.id.120 | 10 | Inverse variance weighted | 0.03 | 0.05 | 1.03 (0.94, 1.13) | 0.522 |
|  |  |  | MR Egger | 0.16 | 0.20 | 1.17 (0.79, 1.74) | 0.451 |
|  |  |  | Simple mode | 0.06 | 0.09 | 1.06 (0.88, 1.27) | 0.567 |
|  |  |  | Weighted median | 0.05 | 0.06 | 1.05 (0.94, 1.17) | 0.357 |
|  |  |  | Weighted mode | 0.06 | 0.10 | 1.06 (0.88, 1.28) | 0.574 |
|  | MollicutesRF9.id.11579 | 12 | Inverse variance weighted | -0.04 | 0.06 | 0.97 (0.85, 1.09) | 0.570 |
|  |  |  | MR Egger | 0.10 | 0.19 | 1.11 (0.76, 1.62) | 0.612 |
|  |  |  | Simple mode | -0.06 | 0.12 | 0.94 (0.74, 1.18) | 0.599 |
|  |  |  | Weighted median | -0.03 | 0.08 | 0.97 (0.83, 1.13) | 0.674 |
|  |  |  | Weighted mode | -0.05 | 0.11 | 0.95 (0.77, 1.17) | 0.636 |
|  | NB1n.id.3953 | 15 | Inverse variance weighted | -0.03 | 0.04 | 0.97 (0.89, 1.05) | 0.476 |
|  |  |  | MR Egger | -0.09 | 0.17 | 0.91 (0.65, 1.28) | 0.601 |
|  |  |  | Simple mode | -0.01 | 0.10 | 0.99 (0.81, 1.21) | 0.925 |
|  |  |  | Weighted median | -0.01 | 0.06 | 0.99 (0.88, 1.10) | 0.824 |
|  |  |  | Weighted mode | -0.03 | 0.10 | 0.97 (0.80, 1.18) | 0.779 |
|  | Pasteurellales.id.3688 | 15 | Inverse variance weighted | -0.02 | 0.05 | 0.98 (0.88, 1.08) | 0.674 |
|  |  |  | MR Egger | 0.06 | 0.12 | 1.06 (0.84, 1.35) | 0.610 |
|  |  |  | Simple mode | -0.08 | 0.12 | 0.93 (0.74, 1.17) | 0.532 |
|  |  |  | Weighted median | -0.01 | 0.07 | 0.99 (0.86, 1.13) | 0.847 |
|  |  |  | Weighted mode | -0.03 | 0.10 | 0.97 (0.79, 1.18) | 0.738 |
|  | Rhodospirillales.id.2667 | 14 | Inverse variance weighted | 0.00 | 0.05 | 1.00 (0.90, 1.12) | 0.933 |
|  |  |  | MR Egger | 0.18 | 0.24 | 1.20 (0.75, 1.94) | 0.465 |
|  |  |  | Simple mode | 0.07 | 0.13 | 1.07 (0.84, 1.37) | 0.585 |
|  |  |  | Weighted median | 0.04 | 0.07 | 1.04 (0.90, 1.20) | 0.620 |
|  |  |  | Weighted mode | 0.07 | 0.13 | 1.07 (0.84, 1.37) | 0.587 |
|  | Selenomonadales.id.2165 | 12 | Inverse variance weighted | 0.04 | 0.12 | 1.04 (0.83, 1.31) | 0.720 |
|  |  |  | MR Egger | 0.13 | 0.39 | 1.14 (0.53, 2.42) | 0.747 |
|  |  |  | Simple mode | 0.13 | 0.23 | 1.13 (0.72, 1.78) | 0.599 |
|  |  |  | Weighted median | -0.04 | 0.13 | 0.97 (0.75, 1.24) | 0.780 |
|  |  |  | Weighted mode | 0.03 | 0.20 | 1.03 (0.70, 1.51) | 0.898 |
|  | Verrucomicrobiales.id.4030 | 11 | Inverse variance weighted | 0.03 | 0.07 | 1.03 (0.90, 1.17) | 0.680 |
|  |  |  | MR Egger | -0.04 | 0.25 | 0.96 (0.59, 1.56) | 0.875 |
|  |  |  | Simple mode | 0.08 | 0.14 | 1.08 (0.82, 1.42) | 0.583 |
|  |  |  | Weighted median | 0.05 | 0.09 | 1.06 (0.88, 1.26) | 0.552 |
|  |  |  | Weighted mode | 0.07 | 0.13 | 1.07 (0.84, 1.37) | 0.597 |
|  | Victivallales.id.2254 | 8 | Inverse variance weighted | -0.05 | 0.05 | 0.95 (0.86, 1.05) | 0.340 |
|  |  |  | MR Egger | -0.21 | 0.17 | 0.81 (0.58, 1.14) | 0.272 |
|  |  |  | Simple mode | -0.02 | 0.09 | 0.98 (0.81, 1.17) | 0.803 |
|  |  |  | Weighted median | -0.04 | 0.06 | 0.96 (0.86, 1.09) | 0.551 |
|  |  |  | Weighted mode | -0.02 | 0.09 | 0.98 (0.81, 1.18) | 0.811 |
| Family | Acidaminococcaceae.id.2166 | 8 | Inverse variance weighted | 0.09 | 0.08 | 1.09 (0.93, 1.28) | 0.292 |
|  |  |  | MR Egger | 0.35 | 0.24 | 1.42 (0.89, 2.27) | 0.192 |
|  |  |  | Simple mode | 0.04 | 0.17 | 1.04 (0.75, 1.45) | 0.802 |
|  |  |  | Weighted median | 0.05 | 0.11 | 1.05 (0.85, 1.31) | 0.648 |
|  |  |  | Weighted mode | 0.03 | 0.16 | 1.04 (0.76, 1.41) | 0.834 |
|  | Actinomycetaceae.id.421 | 5 | Inverse variance weighted | 0.08 | 0.08 | 1.08 (0.93, 1.26) | 0.328 |
|  |  |  | MR Egger | -0.12 | 0.20 | 0.89 (0.60, 1.31) | 0.590 |
|  |  |  | Simple mode | -0.02 | 0.15 | 0.98 (0.74, 1.30) | 0.891 |
|  |  |  | Weighted median | 0.01 | 0.10 | 1.01 (0.82, 1.24) | 0.914 |
|  |  |  | Weighted mode | -0.03 | 0.13 | 0.97 (0.75, 1.26) | 0.845 |
|  | Alcaligenaceae.id.2875 | 11 | Inverse variance weighted | 0.04 | 0.10 | 1.04 (0.86, 1.27) | 0.661 |
|  |  |  | MR Egger | 0.36 | 0.45 | 1.44 (0.60, 3.45) | 0.437 |
|  |  |  | Simple mode | 0.31 | 0.24 | 1.36 (0.86, 2.17) | 0.217 |
|  |  |  | Weighted median | 0.04 | 0.12 | 1.04 (0.82, 1.32) | 0.728 |
|  |  |  | Weighted mode | 0.30 | 0.24 | 1.35 (0.84, 2.16) | 0.244 |
|  | Bacteroidaceae.id.917 | 10 | Inverse variance weighted | 0.05 | 0.09 | 1.05 (0.88, 1.26) | 0.553 |
|  |  |  | MR Egger | 0.15 | 0.50 | 1.16 (0.44, 3.08) | 0.771 |
|  |  |  | Simple mode | 0.21 | 0.21 | 1.24 (0.82, 1.86) | 0.335 |
|  |  |  | Weighted median | 0.10 | 0.19 | 1.11 (0.88, 1.40) | 0.395 |
|  |  |  | Weighted mode | 0.21 | 0.21 | 1.23 (0.82, 1.86) | 0.345 |
|  | BacteroidalesS24.7group.id.11173 | 8 | Inverse variance weighted | 0.03 | 0.07 | 1.03 (0.89, 1.19) | 0.700 |
|  |  |  | MR Egger | 0.58 | 0.29 | 1.79 (1.01, 3.18) | 0.095 |
|  |  |  | Simple mode | -0.10 | 0.13 | 0.90 (0.70, 1.17) | 0.464 |
|  |  |  | Weighted median | -0.06 | 0.09 | 0.94 (0.79, 1.12) | 0.497 |
|  |  |  | Weighted mode | -0.10 | 0.13 | 0.90 (0.70, 1.17) | 0.457 |
|  | Bifidobacteriaceae.id.433 | 11 | Inverse variance weighted | -0.05 | 0.09 | 0.95 (0.82, 1.13) | 0.551 |
|  |  |  | MR Egger | -0.01 | 0.42 | 0.99 (0.43, 2.26) | 0.977 |
|  |  |  | Simple mode | -0.24 | 0.20 | 0.78 (0.53, 1.17) | 0.259 |
|  |  |  | Weighted median | -0.17 | 0.12 | 0.85 (0.67, 1.06) | 0.150 |
|  |  |  | Weighted mode | -0.24 | 0.19 | 0.79 (0.54, 1.15) | 0.240 |
|  | Christensenellaceae.id.1866 | 12 | Inverse variance weighted | 0.11 | 0.07 | 1.11 (0.96, 1.29) | 0.146 |
|  |  |  | MR Egger | 0.20 | 0.16 | 1.22 (0.89, 1.68) | 0.253 |
|  |  |  | Simple mode | 0.19 | 0.16 | 1.21 (0.89, 1.66) | 0.248 |
|  |  |  | Weighted median | 0.13 | 0.10 | 1.14 (0.93, 1.40) | 0.203 |
|  |  |  | Weighted mode | 0.16 | 0.14 | 1.17 (0.89, 1.54) | 0.280 |
|  | Clostridiaceae1.id.1869 | 10 | Inverse variance weighted | 0.21 | 0.09 | 1.24 (1.03, 1.48) | 0.023 |
|  |  |  | MR Egger | 0.22 | 0.28 | 1.25 (0.72, 2.17) | 0.448 |
|  |  |  | Simple mode | 0.24 | 0.19 | 1.27 (0.87, 1.85) | 0.249 |
|  |  |  | Weighted median | 0.20 | 0.12 | 1.22 (0.98, 1.54) | 0.079 |
|  |  |  | Weighted mode | 0.21 | 0.16 | 1.24 (0.91, 1.68) | 0.210 |
|  | ClostridialesvadinBB60group.id.11286 | 15 | Inverse variance weighted | -0.01 | 0.07 | 0.99 (0.86, 1.14) | 0.929 |
|  |  |  | MR Egger | 0.22 | 0.20 | 1.24 (0.84, 1.83) | 0.294 |
|  |  |  | Simple mode | -0.04 | 0.13 | 0.96 (0.74, 1.24) | 0.754 |
|  |  |  | Weighted median | -0.01 | 0.08 | 0.99 (0.85, 1.16) | 0.930 |
|  |  |  | Weighted mode | -0.02 | 0.12 | 0.98 (0.77, 1.24) | 0.873 |
|  | Coriobacteriaceae.id.811 | 13 | Inverse variance weighted | -0.06 | 0.08 | 0.94 (0.80, 1.10) | 0.441 |
|  |  |  | MR Egger | 0.17 | 0.32 | 1.19 (0.63, 2.22) | 0.601 |
|  |  |  | Simple mode | -0.14 | 0.17 | 0.87 (0.62, 1.22) | 0.433 |
|  |  |  | Weighted median | -0.08 | 0.11 | 0.92 (0.74, 1.14) | 0.435 |
|  |  |  | Weighted mode | -0.13 | 0.16 | 0.88 (0.64, 1.21) | 0.451 |
|  | Defluviitaleaceae.id.1924 | 11 | Inverse variance weighted | 0.00 | 0.06 | 1.00 (0.90, 1.12) | 0.995 |
|  |  |  | MR Egger | 0.00 | 0.18 | 1.00 (0.70, 1.44) | 0.991 |
|  |  |  | Simple mode | -0.01 | 0.11 | 0.99 (0.79, 1.24) | 0.910 |
|  |  |  | Weighted median | 0.00 | 0.07 | 1.00 (0.87, 1.16) | 0.979 |
|  |  |  | Weighted mode | -0.02 | 0.11 | 0.98 (0.80, 1.21) | 0.866 |
|  | Desulfovibrionaceae.id.3169 | 10 | Inverse variance weighted | -0.12 | 0.08 | 0.89 (0.76, 1.03) | 0.118 |
|  |  |  | MR Egger | -0.08 | 0.19 | 0.92 (0.63, 1.34) | 0.679 |
|  |  |  | Simple mode | -0.25 | 0.16 | 0.78 (0.57, 1.08) | 0.167 |
|  |  |  | Weighted median | -0.14 | 0.10 | 0.87 (0.72, 1.06) | 0.172 |
|  |  |  | Weighted mode | -0.18 | 0.14 | 0.84 (0.63, 1.11) | 0.250 |
|  | Enterobacteriaceae.id.3469 | 6 | Inverse variance weighted | 0.20 | 0.11 | 1.22 (0.99, 1.51) | 0.057 |
|  |  |  | MR Egger | -0.25 | 0.63 | 0.78 (0.23, 2.67) | 0.710 |
|  |  |  | Simple mode | 0.23 | 0.21 | 1.26 (0.84, 1.89) | 0.321 |
|  |  |  | Weighted median | 0.23 | 0.13 | 1.25 (0.96, 1.63) | 0.092 |
|  |  |  | Weighted mode | 0.23 | 0.21 | 1.25 (0.82, 1.91) | 0.338 |
|  | Erysipelotrichaceae.id.2149 | 13 | Inverse variance weighted | 0.04 | 0.09 | 1.04 (0.88, 1.23) | 0.642 |
|  |  |  | MR Egger | -0.04 | 0.38 | 0.96 (0.46, 2.02) | 0.920 |
|  |  |  | Simple mode | -0.06 | 0.20 | 0.94 (0.64, 1.40) | 0.772 |
|  |  |  | Weighted median | 0.03 | 0.11 | 1.03 (0.82, 1.29) | 0.809 |
|  |  |  | Weighted mode | -0.03 | 0.19 | 0.97 (0.67, 1.40) | 0.861 |
|  | FamilyXI.id.1936 | 8 | Inverse variance weighted | 0.02 | 0.06 | 1.02 (0.92, 1.14) | 0.684 |
|  |  |  | MR Egger | 0.30 | 0.36 | 1.36 (0.67, 2.76) | 0.433 |
|  |  |  | Simple mode | -0.03 | 0.12 | 0.97 (0.76, 1.24) | 0.824 |
|  |  |  | Weighted median | -0.00 | 0.06 | 1.00 (0.89, 1.12) | 0.991 |
|  |  |  | Weighted mode | -0.03 | 0.12 | 0.97 (0.77, 1.22) | 0.811 |
|  | FamilyXIII.id.1957 | 11 | Inverse variance weighted | -0.07 | 0.08 | 0.93 (0.79, 1.09) | 0.376 |
|  |  |  | MR Egger | -0.17 | 0.29 | 0.85 (0.48, 1.50) | 0.581 |
|  |  |  | Simple mode | -0.10 | 0.18 | 0.91 (0.64, 1.28) | 0.593 |
|  |  |  | Weighted median | -0.09 | 0.11 | 0.91 (0.73, 1.14) | 0.423 |
|  |  |  | Weighted mode | -0.10 | 0.16 | 0.91 (0.66, 1.25) | 0.568 |
|  | Lachnospiraceae.id.1987 | 17 | Inverse variance weighted | 0.05 | 0.07 | 1.05 (0.91, 1.20) | 0.518 |
|  |  |  | MR Egger | 0.01 | 0.18 | 1.01 (0.71, 1.44) | 0.936 |
|  |  |  | Simple mode | 0.01 | 0.16 | 1.01 (0.73, 1.39) | 0.949 |
|  |  |  | Weighted median | -0.00 | 0.10 | 1.00 (0.82, 1.21) | 0.990 |
|  |  |  | Weighted mode | -0.01 | 0.14 | 0.99 (0.75, 1.31) | 0.944 |
|  | Lactobacillaceae.id.1836 | 8 | Inverse variance weighted | -0.11 | 0.06 | 0.90 (0.80, 1.00) | 0.059 |
|  |  |  | MR Egger | -0.27 | 0.15 | 0.76 (0.56, 1.03) | 0.126 |
|  |  |  | Simple mode | -0.05 | 0.11 | 0.95 (0.77, 1.17) | 0.661 |
|  |  |  | Weighted median | -0.11 | 0.07 | 0.90 (0.78, 1.03) | 0.136 |
|  |  |  | Weighted mode | -0.08 | 0.09 | 0.92 (0.77, 1.11) | 0.419 |
|  | Methanobacteriaceae.id.121 | 10 | Inverse variance weighted | 0.03 | 0.05 | 1.03 (0.94, 1.13) | 0.522 |
|  |  |  | MR Egger | 0.16 | 0.20 | 1.17 (0.79, 1.74) | 0.451 |
|  |  |  | Simple mode | 0.06 | 0.09 | 1.06 (0.88, 1.27) | 0.555 |
|  |  |  | Weighted median | 0.05 | 0.06 | 1.05 (0.94, 1.18) | 0.363 |
|  |  |  | Weighted mode | 0.06 | 0.10 | 1.06 (0.88, 1.28) | 0.573 |
|  | Oxalobacteraceae.id.2966 | 14 | Inverse variance weighted | -0.03 | 0.04 | 0.97 (0.90, 1.05) | 0.449 |
|  |  |  | MR Egger | -0.08 | 0.16 | 0.92 (0.67, 1.26) | 0.613 |
|  |  |  | Simple mode | 0.02 | 0.09 | 1.02 (0.85, 1.23) | 0.809 |
|  |  |  | Weighted median | 0.00 | 0.05 | 1.00 (0.90, 1.12) | 0.978 |
|  |  |  | Weighted mode | 0.03 | 0.09 | 1.03 (0.86, 1.23) | 0.763 |
|  | Pasteurellaceae.id.3689 | 15 | Inverse variance weighted | -0.02 | 0.05 | 0.98 (0.88, 1.08) | 0.674 |
|  |  |  | MR Egger | 0.06 | 0.12 | 1.06 (0.84, 1.35) | 0.610 |
|  |  |  | Simple mode | -0.08 | 0.13 | 0.93 (0.72, 1.19) | 0.565 |
|  |  |  | Weighted median | -0.01 | 0.07 | 0.99 (0.86, 1.13) | 0.844 |
|  |  |  | Weighted mode | -0.03 | 0.11 | 0.97 (0.78, 1.19) | 0.751 |
|  | Peptococcaceae.id.2024 | 8 | Inverse variance weighted | 0.03 | 0.08 | 1.04 (0.89, 1.20) | 0.656 |
|  |  |  | MR Egger | 0.05 | 0.19 | 1.05 (0.72, 1.54) | 0.806 |
|  |  |  | Simple mode | 0.02 | 0.14 | 1.02 (0.77, 1.35) | 0.912 |
|  |  |  | Weighted median | 0.09 | 0.09 | 1.10 (0.92, 1.31) | 0.318 |
|  |  |  | Weighted mode | 0.10 | 0.11 | 1.11 (0.89, 1.38) | 0.402 |
|  | Peptostreptococcaceae.id.2042 | 13 | Inverse variance weighted | -0.02 | 0.06 | 0.98 (0.86, 1.11) | 0.712 |
|  |  |  | MR Egger | -0.19 | 0.14 | 0.82 (0.63, 1.08) | 0.185 |
|  |  |  | Simple mode | -0.10 | 0.13 | 0.91 (0.70, 1.17) | 0.479 |
|  |  |  | Weighted median | -0.10 | 0.08 | 0.91 (0.77, 1.06) | 0.231 |
|  |  |  | Weighted mode | -0.12 | 0.11 | 0.89 (0.71, 1.10) | 0.306 |
|  | Porphyromonadaceae.id.943 | 9 | Inverse variance weighted | 0.17 | 0.10 | 1.19 (0.97, 1.45) | 0.096 |
|  |  |  | MR Egger | 0.27 | 0.46 | 1.31 (0.54, 3.20) | 0.570 |
|  |  |  | Simple mode | 0.02 | 0.22 | 1.02 (0.66, 1.57) | 0.938 |
|  |  |  | Weighted median | 0.08 | 0.14 | 1.08 (0.83, 1.41) | 0.574 |
|  |  |  | Weighted mode | 0.03 | 0.19 | 1.03 (0.70, 1.50) | 0.898 |
|  | Prevotellaceae.id.960 | 16 | Inverse variance weighted | 0.02 | 0.06 | 1.02 (0.90, 1.15) | 0.776 |
|  |  |  | MR Egger | 0.06 | 0.22 | 1.06 (0.68, 1.64) | 0.801 |
|  |  |  | Simple mode | 0.04 | 0.16 | 1.04 (0.76, 1.43) | 0.791 |
|  |  |  | Weighted median | 0.03 | 0.09 | 1.03 (0.87, 1.22) | 0.750 |
|  |  |  | Weighted mode | 0.06 | 0.15 | 1.07 (0.80, 1.42) | 0.668 |
|  | Rhodospirillaceae.id.2717 | 15 | Inverse variance weighted | -0.01 | 0.05 | 0.99 (0.89, 1.09) | 0.782 |
|  |  |  | MR Egger | 0.03 | 0.25 | 1.03 (0.64, 1.68) | 0.898 |
|  |  |  | Simple mode | -0.01 | 0.12 | 0.99 (0.78, 1.24) | 0.907 |
|  |  |  | Weighted median | -0.03 | 0.07 | 0.97 (0.85, 1.11) | 0.671 |
|  |  |  | Weighted mode | -0.02 | 0.13 | 0.98 (0.77, 1.25) | 0.871 |
|  | Rikenellaceae.id.967 | 19 | Inverse variance weighted | 0.02 | 0.06 | 1.02 (0.90, 1.16) | 0.733 |
|  |  |  | MR Egger | -0.01 | 0.19 | 0.99 (0.68, 1.45) | 0.974 |
|  |  |  | Simple mode | 0.14 | 0.14 | 1.15 (0.87, 1.53) | 0.332 |
|  |  |  | Weighted median | 0.06 | 0.09 | 1.06 (0.89, 1.26) | 0.493 |
|  |  |  | Weighted mode | 0.12 | 0.14 | 1.13 (0.87, 1.48) | 0.368 |
|  | Ruminococcaceae.id.2050 | 10 | Inverse variance weighted | 0.03 | 0.09 | 1.03 (0.87, 1.22) | 0.725 |
|  |  |  | MR Egger | 0.16 | 0.19 | 1.17 (0.81, 1.69) | 0.425 |
|  |  |  | Simple mode | 0.21 | 0.16 | 1.23 (0.90, 1.68) | 0.223 |
|  |  |  | Weighted median | 0.15 | 0.11 | 1.16 (0.93, 1.44) | 0.192 |
|  |  |  | Weighted mode | 0.18 | 0.14 | 1.20 (0.92, 1.56) | 0.219 |
|  | Streptococcaceae.id.1850 | 11 | Inverse variance weighted | 0.06 | 0.08 | 1.06 (0.90, 1.25) | 0.464 |
|  |  |  | MR Egger | 0.38 | 0.32 | 1.46 (0.78, 2.71) | 0.266 |
|  |  |  | Simple mode | -0.07 | 0.18 | 0.94 (0.66, 1.32) | 0.714 |
|  |  |  | Weighted median | 0.01 | 0.11 | 1.01 (0.81, 1.24) | 0.955 |
|  |  |  | Weighted mode | -0.07 | 0.19 | 0.93 (0.65, 1.35) | 0.722 |
|  | unknownfamily.id.1000001214 | 9 | Inverse variance weighted | -0.03 | 0.08 | 0.97 (0.83, 1.14) | 0.739 |
|  |  |  | MR Egger | 0.27 | 0.20 | 1.31 (0.88, 1.96) | 0.221 |
|  |  |  | Simple mode | -0.23 | 0.17 | 0.80 (0.57, 1.12) | 0.227 |
|  |  |  | Weighted median | 0.01 | 0.08 | 1.01 (0.86, 1.18) | 0.927 |
|  |  |  | Weighted mode | 0.09 | 0.15 | 1.10 (0.82, 1.47) | 0.554 |
|  | unknownfamily.id.1000005471 | 12 | Inverse variance weighted | -0.04 | 0.06 | 0.97 (0.85, 1.09) | 0.570 |
|  |  |  | MR Egger | 0.10 | 0.19 | 1.11 (0.76, 1.62) | 0.612 |
|  |  |  | Simple mode | -0.06 | 0.12 | 0.94 (0.74, 1.18) | 0.593 |
|  |  |  | Weighted median | -0.03 | 0.08 | 0.97 (0.82, 1.14) | 0.686 |
|  |  |  | Weighted mode | -0.05 | 0.11 | 0.95 (0.77, 1.17) | 0.643 |
|  | unknownfamily.id.1000006161 | 15 | Inverse variance weighted | -0.03 | 0.04 | 0.97 (0.89, 1.05) | 0.476 |
|  |  |  | MR Egger | -0.09 | 0.17 | 0.91 (0.65, 1.28) | 0.601 |
|  |  |  | Simple mode | -0.01 | 0.11 | 0.99 (0.80, 1.23) | 0.931 |
|  |  |  | Weighted median | -0.01 | 0.06 | 0.99 (0.89, 1.10) | 0.822 |
|  |  |  | Weighted mode | -0.03 | 0.09 | 0.97 (0.81, 1.16) | 0.760 |
|  | Veillonellaceae.id.2172 | 19 | Inverse variance weighted | -0.08 | 0.06 | 0.93 (0.83, 1.03) | 0.175 |
|  |  |  | MR Egger | -0.04 | 0.13 | 0.96 (0.75, 1.23) | 0.736 |
|  |  |  | Simple mode | -0.17 | 0.14 | 0.84 (0.64, 1.11) | 0.234 |
|  |  |  | Weighted median | -0.09 | 0.08 | 0.91 (0.78, 1.07) | 0.251 |
|  |  |  | Weighted mode | -0.11 | 0.11 | 0.90 (0.73, 1.11) | 0.322 |
|  | Verrucomicrobiaceae.id.4036 | 11 | Inverse variance weighted | 0.03 | 0.07 | 1.03 (0.90, 1.17) | 0.681 |
|  |  |  | MR Egger | -0.04 | 0.25 | 0.96 (0.59, 1.56) | 0.871 |
|  |  |  | Simple mode | 0.08 | 0.14 | 1.08 (0.83, 1.41) | 0.571 |
|  |  |  | Weighted median | 0.05 | 0.09 | 1.06 (0.88, 1.26) | 0.547 |
|  |  |  | Weighted mode | 0.07 | 0.14 | 1.07 (0.82, 1.40) | 0.621 |
|  | Victivallaceae.id.2255 | 12 | Inverse variance weighted | -0.01 | 0.04 | 0.99 (0.92, 1.06) | 0.725 |
|  |  |  | MR Egger | -0.04 | 0.17 | 0.96 (0.69, 1.32) | 0.794 |
|  |  |  | Simple mode | -0.01 | 0.07 | 0.99 (0.86, 1.14) | 0.910 |
|  |  |  | Weighted median | -0.01 | 0.05 | 0.99 (0.90, 1.08) | 0.789 |
|  |  |  | Weighted mode | -0.01 | 0.07 | 0.99 (0.86, 1.15) | 0.912 |
| Genus | Clostridiuminnocuumgroup.id.14397 | 9 | Inverse variance weighted | -0.07 | 0.05 | 0.93 (0.84, 1.03) | 0.178 |
|  |  |  | MR Egger | 0.03 | 0.28 | 1.03 (0.60, 1.79) | 0.906 |
|  |  |  | Simple mode | -0.02 | 0.10 | 0.98 (0.80, 1.20) | 0.836 |
|  |  |  | Weighted median | -0.06 | 0.07 | 0.95 (0.83, 1.08) | 0.401 |
|  |  |  | Weighted mode | -0.02 | 0.09 | 0.98 (0.81, 1.18) | 0.805 |
|  | Eubacteriumbrachygroup.id.11296 | 10 | Inverse variance weighted | 0.01 | 0.04 | 1.01 (0.93, 1.10) | 0.803 |
|  |  |  | MR Egger | 0.01 | 0.17 | 1.01 (0.73, 1.40) | 0.951 |
|  |  |  | Simple mode | 0.02 | 0.09 | 1.02 (0.86, 1.21) | 0.845 |
|  |  |  | Weighted median | 0.01 | 0.05 | 1.01 (0.91, 1.13) | 0.799 |
|  |  |  | Weighted mode | 0.02 | 0.10 | 1.02 (0.84, 1.23) | 0.865 |
|  | Eubacteriumcoprostanoligenesgroup.id.11375 | 13 | Inverse variance weighted | -0.07 | 0.09 | 0.94 (0.79, 1.11) | 0.450 |
|  |  |  | MR Egger | -0.12 | 0.37 | 0.89 (0.43, 1.82) | 0.749 |
|  |  |  | Simple mode | -0.09 | 0.19 | 0.91 (0.62, 1.34) | 0.650 |
|  |  |  | Weighted median | -0.08 | 0.11 | 0.93 (0.75, 1.15) | 0.486 |
|  |  |  | Weighted mode | -0.12 | 0.18 | 0.89 (0.62, 1.27) | 0.519 |
|  | Eubacteriumeligensgroup.id.14372 | 7 | Inverse variance weighted | 0.08 | 0.10 | 1.08 (0.89, 1.30) | 0.434 |
|  |  |  | MR Egger | 0.36 | 0.39 | 1.43 (0.67, 3.06) | 0.401 |
|  |  |  | Simple mode | 0.15 | 0.19 | 1.16 (0.79, 1.70) | 0.474 |
|  |  |  | Weighted median | 0.11 | 0.13 | 1.12 (0.87, 1.44) | 0.375 |
|  |  |  | Weighted mode | 0.17 | 0.18 | 1.18 (0.83, 1.69) | 0.397 |
|  | Eubacteriumfissicatenagroup.id.14373 | 9 | Inverse variance weighted | -0.02 | 0.04 | 0.98 (0.90, 1.07) | 0.634 |
|  |  |  | MR Egger | 0.05 | 0.24 | 1.05 (0.66, 1.68) | 0.831 |
|  |  |  | Simple mode | 0.02 | 0.09 | 1.02 (0.86, 1.22) | 0.791 |
|  |  |  | Weighted median | 0.00 | 0.06 | 1.00 (0.90, 1.12) | 0.982 |
|  |  |  | Weighted mode | 0.03 | 0.08 | 1.03 (0.88, 1.21) | 0.730 |
|  | Eubacteriumhalliigroup.id.11338 | 16 | Inverse variance weighted | -0.05 | 0.06 | 0.95 (0.85, 1.07) | 0.429 |
|  |  |  | MR Egger | -0.03 | 0.13 | 0.97 (0.76, 1.24) | 0.793 |
|  |  |  | Simple mode | 0.04 | 0.13 | 1.04 (0.81, 1.36) | 0.746 |
|  |  |  | Weighted median | -0.00 | 0.08 | 1.00 (0.85, 1.17) | 0.978 |
|  |  |  | Weighted mode | 0.03 | 0.13 | 1.03 (0.81, 1.32) | 0.791 |
|  | Eubacteriumnodatumgroup.id.11297 | 11 | Inverse variance weighted | -0.00 | 0.04 | 1.00 (0.91, 1.09) | 0.970 |
|  |  |  | MR Egger | -0.33 | 0.17 | 0.72 (0.51, 1.01) | 0.090 |
|  |  |  | Simple mode | 0.03 | 0.10 | 1.03 (0.85, 1.25) | 0.784 |
|  |  |  | Weighted median | 0.00 | 0.05 | 1.00 (0.91, 1.11) | 0.938 |
|  |  |  | Weighted mode | 0.04 | 0.09 | 1.04 (0.87, 1.24) | 0.707 |
|  | Eubacteriumoxidoreducensgroup.id.11339 | 4 | Inverse variance weighted | 0.07 | 0.09 | 1.07 (0.90, 1.27) | 0.427 |
|  |  |  | MR Egger | 0.13 | 0.32 | 1.14 (0.61, 2.11) | 0.725 |
|  |  |  | Simple mode | 0.19 | 0.14 | 1.21 (0.91, 1.61) | 0.277 |
|  |  |  | Weighted median | 0.10 | 0.09 | 1.11 (0.92, 1.33) | 0.270 |
|  |  |  | Weighted mode | 0.17 | 0.13 | 1.19 (0.92, 1.53) | 0.283 |
|  | Eubacteriumrectalegroup.id.14374 | 8 | Inverse variance weighted | 0.10 | 0.12 | 1.11 (0.88, 1.39) | 0.378 |
|  |  |  | MR Egger | -0.75 | 0.35 | 0.47 (0.24, 0.93) | 0.073 |
|  |  |  | Simple mode | -0.11 | 0.26 | 0.90 (0.54, 1.50) | 0.693 |
|  |  |  | Weighted median | 0.03 | 0.14 | 1.03 (0.78, 1.36) | 0.842 |
|  |  |  | Weighted mode | -0.12 | 0.21 | 0.89 (0.59, 1.34) | 0.584 |
|  | Eubacteriumruminantiumgroup.id.11340 | 18 | Inverse variance weighted | -0.02 | 0.05 | 0.98 (0.90, 1.08) | 0.708 |
|  |  |  | MR Egger | 0.19 | 0.16 | 1.21 (0.88, 1.67) | 0.247 |
|  |  |  | Simple mode | 0.08 | 0.11 | 1.08 (0.87, 1.35) | 0.489 |
|  |  |  | Weighted median | 0.04 | 0.06 | 1.04 (0.91, 1.18) | 0.562 |
|  |  |  | Weighted mode | 0.08 | 0.12 | 1.08 (0.86, 1.36) | 0.513 |
|  | Eubacteriumventriosumgroup.id.11341 | 13 | Inverse variance weighted | 0.15 | 0.09 | 1.16 (0.97, 1.39) | 0.114 |
|  |  |  | MR Egger | 0.44 | 0.41 | 1.55 (0.70, 3.45) | 0.302 |
|  |  |  | Simple mode | -0.12 | 0.23 | 0.89 (0.56, 1.40) | 0.617 |
|  |  |  | Weighted median | 0.14 | 0.11 | 1.15 (0.92, 1.44) | 0.220 |
|  |  |  | Weighted mode | -0.10 | 0.22 | 0.90 (0.59, 1.39) | 0.656 |
|  | Eubacteriumxylanophilumgroup.id.14375 | 9 | Inverse variance weighted | -0.08 | 0.11 | 0.93 (0.75, 1.15) | 0.483 |
|  |  |  | MR Egger | -0.39 | 0.33 | 0.68 (0.36, 1.28) | 0.272 |
|  |  |  | Simple mode | 0.06 | 0.20 | 1.06 (0.72, 1.56) | 0.765 |
|  |  |  | Weighted median | -0.01 | 0.11 | 0.99 (0.80, 1.24) | 0.954 |
|  |  |  | Weighted mode | 0.06 | 0.19 | 1.06 (0.73, 1.55) | 0.763 |
|  | Ruminococcusgauvreauiigroup.id.11342 | 12 | Inverse variance weighted | 0.08 | 0.07 | 1.08 (0.94, 1.25) | 0.268 |
|  |  |  | MR Egger | 0.25 | 0.32 | 1.29 (0.69, 2.41) | 0.450 |
|  |  |  | Simple mode | 0.13 | 0.15 | 1.13 (0.85, 1.51) | 0.407 |
|  |  |  | Weighted median | 0.10 | 0.10 | 1.10 (0.92, 1.33) | 0.299 |
|  |  |  | Weighted mode | 0.11 | 0.15 | 1.12 (0.83, 1.51) | 0.472 |
|  | Ruminococcusgnavusgroup.id.14376 | 12 | Inverse variance weighted | -0.04 | 0.05 | 0.96 (0.87, 1.07) | 0.482 |
|  |  |  | MR Egger | 0.07 | 0.25 | 1.08 (0.66, 1.76) | 0.780 |
|  |  |  | Simple mode | -0.08 | 0.12 | 0.92 (0.73, 1.17) | 0.529 |
|  |  |  | Weighted median | -0.05 | 0.07 | 0.95 (0.83, 1.09) | 0.455 |
|  |  |  | Weighted mode | -0.06 | 0.12 | 0.94 (0.75, 1.18) | 0.589 |
|  | Ruminococcustorquesgroup.id.14377 | 9 | Inverse variance weighted | -0.10 | 0.09 | 0.90 (0.76, 1.07) | 0.250 |
|  |  |  | MR Egger | 0.08 | 0.23 | 1.09 (0.70, 1.70) | 0.727 |
|  |  |  | Simple mode | -0.07 | 0.19 | 0.94 (0.64, 1.36) | 0.740 |
|  |  |  | Weighted median | -0.11 | 0.12 | 0.90 (0.71, 1.13) | 0.364 |
|  |  |  | Weighted mode | -0.09 | 0.18 | 0.92 (0.64, 1.31) | 0.649 |
|  | Actinomyces.id.423 | 7 | Inverse variance weighted | 0.03 | 0.07 | 1.03 (0.90, 1.18) | 0.667 |
|  |  |  | MR Egger | 0.07 | 0.21 | 1.07 (0.72, 1.61) | 0.748 |
|  |  |  | Simple mode | 0.14 | 0.14 | 1.15 (0.87, 1.52) | 0.366 |
|  |  |  | Weighted median | 0.02 | 0.09 | 1.02 (0.86, 1.22) | 0.790 |
|  |  |  | Weighted mode | 0.03 | 0.14 | 1.03 (0.79, 1.34) | 0.848 |
|  | Adlercreutzia.id.812 | 8 | Inverse variance weighted | -0.02 | 0.07 | 0.98 (0.86, 1.13) | 0.820 |
|  |  |  | MR Egger | -0.02 | 0.32 | 0.98 (0.53, 1.83) | 0.952 |
|  |  |  | Simple mode | -0.06 | 0.12 | 0.94 (0.74, 1.20) | 0.634 |
|  |  |  | Weighted median | -0.02 | 0.08 | 0.98 (0.83, 1.16) | 0.826 |
|  |  |  | Weighted mode | -0.05 | 0.12 | 0.95 (0.74, 1.21) | 0.673 |
|  | Akkermansia.id.4037 | 11 | Inverse variance weighted | 0.03 | 0.07 | 1.03 (0.90, 1.17) | 0.683 |
|  |  |  | MR Egger | -0.04 | 0.25 | 0.96 (0.59, 1.56) | 0.865 |
|  |  |  | Simple mode | 0.08 | 0.14 | 1.08 (0.83, 1.42) | 0.574 |
|  |  |  | Weighted median | 0.05 | 0.09 | 1.06 (0.89, 1.25) | 0.537 |
|  |  |  | Weighted mode | 0.07 | 0.14 | 1.07 (0.82, 1.40) | 0.628 |
|  | Alistipes.id.968 | 14 | Inverse variance weighted | -0.00 | 0.08 | 1.00 (0.86, 1.17) | 0.987 |
|  |  |  | MR Egger | -0.31 | 0.38 | 0.73 (0.34, 1.56) | 0.435 |
|  |  |  | Simple mode | 0.02 | 0.18 | 1.02 (0.72, 1.46) | 0.910 |
|  |  |  | Weighted median | 0.02 | 0.11 | 1.02 (0.83, 1.25) | 0.861 |
|  |  |  | Weighted mode | 0.01 | 0.18 | 1.01 (0.72, 1.43) | 0.941 |
|  | Allisonella.id.2174 | 8 | Inverse variance weighted | 0.05 | 0.04 | 1.05 (0.97, 1.15) | 0.237 |
|  |  |  | MR Egger | -0.19 | 0.27 | 0.83 (0.49, 1.40) | 0.503 |
|  |  |  | Simple mode | 0.10 | 0.08 | 1.10 (0.94, 1.29) | 0.275 |
|  |  |  | Weighted median | 0.04 | 0.05 | 1.04 (0.94, 1.16) | 0.466 |
|  |  |  | Weighted mode | -0.00 | 0.08 | 1.00 (0.85, 1.17) | 0.992 |
|  | Alloprevotella.id.961 | 6 | Inverse variance weighted | 0.13 | 0.05 | 1.14 (1.03, 1.25) | 0.010 |
|  |  |  | MR Egger | -0.35 | 0.46 | 0.70 (0.28, 1.74) | 0.488 |
|  |  |  | Simple mode | 0.13 | 0.09 | 1.14 (0.95, 1.37) | 0.211 |
|  |  |  | Weighted median | 0.13 | 0.07 | 1.14 (1.01, 1.29) | 0.036 |
|  |  |  | Weighted mode | 0.14 | 0.09 | 1.15 (0.96, 1.36) | 0.187 |
|  | Anaerofilum.id.2053 | 10 | Inverse variance weighted | -0.07 | 0.06 | 0.93 (0.83, 1.05) | 0.243 |
|  |  |  | MR Egger | -0.58 | 0.26 | 0.56 (0.34, 0.93) | 0.055 |
|  |  |  | Simple mode | -0.11 | 0.12 | 0.90 (0.71, 1.13) | 0.389 |
|  |  |  | Weighted median | -0.09 | 0.07 | 0.92 (0.80, 1.05) | 0.201 |
|  |  |  | Weighted mode | -0.09 | 0.11 | 0.91 (0.73, 1.14) | 0.429 |
|  | Anaerostipes.id.1991 | 11 | Inverse variance weighted | 0.00 | 0.08 | 1.00 (0.85, 1.17) | 0.999 |
|  |  |  | MR Egger | 0.25 | 0.26 | 1.28 (0.77, 2.13) | 0.359 |
|  |  |  | Simple mode | -0.01 | 0.17 | 0.99 (0.70, 1.38) | 0.937 |
|  |  |  | Weighted median | -0.02 | 0.11 | 0.98 (0.80, 1.20) | 0.848 |
|  |  |  | Weighted mode | 0.01 | 0.17 | 1.01 (0.72, 1.40) | 0.972 |
|  | Anaerotruncus.id.2054 | 14 | Inverse variance weighted | 0.14 | 0.07 | 1.15 (0.99, 1.33) | 0.061 |
|  |  |  | MR Egger | -0.04 | 0.24 | 0.96 (0.60, 1.52) | 0.859 |
|  |  |  | Simple mode | 0.11 | 0.16 | 1.11 (0.81, 1.53) | 0.525 |
|  |  |  | Weighted median | 0.10 | 0.10 | 1.11 (0.91, 1.35) | 0.323 |
|  |  |  | Weighted mode | 0.11 | 0.16 | 1.11 (0.81, 1.52) | 0.518 |
|  | Bacteroides.id.918 | 10 | Inverse variance weighted | 0.05 | 0.09 | 1.05 (0.88, 1.26) | 0.553 |
|  |  |  | MR Egger | 0.15 | 0.50 | 1.16 (0.44, 3.08) | 0.771 |
|  |  |  | Simple mode | 0.21 | 0.21 | 1.24 (0.83, 1.85) | 0.331 |
|  |  |  | Weighted median | 0.10 | 0.12 | 1.11 (0.88, 1.39) | 0.383 |
|  |  |  | Weighted mode | 0.21 | 0.22 | 1.23 (0.80, 1.89) | 0.363 |
|  | Barnesiella.id.944 | 14 | Inverse variance weighted | 0.05 | 0.08 | 1.05 (0.90, 1.22) | 0.552 |
|  |  |  | MR Egger | -0.14 | 0.27 | 0.87 (0.51, 1.47) | 0.606 |
|  |  |  | Simple mode | 0.17 | 0.19 | 1.19 (0.83, 1.71) | 0.369 |
|  |  |  | Weighted median | 0.00 | 0.10 | 1.00 (0.83, 1.22) | 0.968 |
|  |  |  | Weighted mode | 0.07 | 0.16 | 1.07 (0.78, 1.47) | 0.690 |
|  | Bifidobacterium.id.436 | 10 | Inverse variance weighted | -0.07 | 0.08 | 0.93 (0.80, 1.09) | 0.381 |
|  |  |  | MR Egger | -0.21 | 0.19 | 0.81 (0.55, 1.19) | 0.311 |
|  |  |  | Simple mode | -0.04 | 0.15 | 0.96 (0.72, 1.28) | 0.782 |
|  |  |  | Weighted median | -0.07 | 0.10 | 0.93 (0.77, 1.13) | 0.483 |
|  |  |  | Weighted mode | -0.08 | 0.14 | 0.93 (0.70, 1.22) | 0.600 |
|  | Bilophila.id.3170 | 17 | Inverse variance weighted | 0.02 | 0.08 | 1.02 (0.88, 1.18) | 0.804 |
|  |  |  | MR Egger | 0.49 | 0.28 | 1.63 (0.94, 2.83) | 0.101 |
|  |  |  | Simple mode | 0.02 | 0.17 | 1.02 (0.74, 1.42) | 0.894 |
|  |  |  | Weighted median | -0.01 | 0.09 | 0.99 (0.83, 1.18) | 0.895 |
|  |  |  | Weighted mode | 0.01 | 0.16 | 1.01 (0.74, 1.38) | 0.952 |
|  | Blautia.id.1992 | 13 | Inverse variance weighted | 0.00 | 0.07 | 1.00 (0.87, 1.15) | 0.977 |
|  |  |  | MR Egger | -0.17 | 0.15 | 0.85 (0.63, 1.14) | 0.294 |
|  |  |  | Simple mode | 0.17 | 0.16 | 1.19 (0.87, 1.62) | 0.309 |
|  |  |  | Weighted median | 0.05 | 0.10 | 1.06 (0.87, 1.28) | 0.570 |
|  |  |  | Weighted mode | 0.12 | 0.14 | 1.13 (0.86, 1.48) | 0.398 |
|  | Butyricicoccus.id.2055 | 8 | Inverse variance weighted | -0.03 | 0.08 | 0.97 (0.82, 1.14) | 0.681 |
|  |  |  | MR Egger | 0.16 | 0.17 | 1.17 (0.84, 1.64) | 0.390 |
|  |  |  | Simple mode | -0.17 | 0.20 | 0.85 (0.58, 1.24) | 0.421 |
|  |  |  | Weighted median | 0.03 | 0.11 | 1.03 (0.83, 1.29) | 0.768 |
|  |  |  | Weighted mode | 0.11 | 0.12 | 1.12 (0.88, 1.42) | 0.397 |
|  | Butyricimonas.id.945 | 17 | Inverse variance weighted | -0.00 | 0.05 | 1.00 (0.90, 1.11) | 0.967 |
|  |  |  | MR Egger | 0.21 | 0.19 | 1.23 (0.85, 1.80) | 0.291 |
|  |  |  | Simple mode | 0.09 | 0.12 | 1.09 (0.87, 1.37) | 0.460 |
|  |  |  | Weighted median | 0.01 | 0.07 | 1.01 (0.88, 1.16) | 0.848 |
|  |  |  | Weighted mode | 0.08 | 0.12 | 1.08 (0.86, 1.37) | 0.513 |
|  | Butyrivibrio.id.1993 | 16 | Inverse variance weighted | -0.03 | 0.03 | 0.97 (0.90, 1.03) | 0.328 |
|  |  |  | MR Egger | -0.15 | 0.15 | 0.86 (0.63, 1.16) | 0.338 |
|  |  |  | Simple mode | -0.02 | 0.08 | 0.98 (0.83, 1.16) | 0.857 |
|  |  |  | Weighted median | -0.02 | 0.04 | 0.98 (0.90, 1.06) | 0.589 |
|  |  |  | Weighted mode | -0.02 | 0.08 | 0.98 (0.83, 1.15) | 0.803 |
|  | CandidatusSoleaferrea.id.11350 | 16 | Inverse variance weighted | -0.01 | 0.04 | 0.99 (0.91, 1.08) | 0.804 |
|  |  |  | MR Egger | 0.04 | 0.17 | 1.04 (0.74, 1.47) | 0.809 |
|  |  |  | Simple mode | 0.07 | 0.09 | 1.07 (0.89, 1.29) | 0.467 |
|  |  |  | Weighted median | 0.04 | 0.06 | 1.04 (0.93, 1.16) | 0.482 |
|  |  |  | Weighted mode | 0.07 | 0.09 | 1.08 (0.89, 1.29) | 0.452 |
|  | Catenibacterium.id.2153 | 5 | Inverse variance weighted | -0.13 | 0.05 | 0.88 (0.79, 0.98) | 0.018 |
|  |  |  | MR Egger | 0.13 | 0.53 | 1.14 (0.40, 3.21) | 0.826 |
|  |  |  | Simple mode | -0.18 | 0.11 | 0.84 (0.68, 1.04) | 0.182 |
|  |  |  | Weighted median | -0.12 | 0.08 | 0.88 (0.76, 1.03) | 0.110 |
|  |  |  | Weighted mode | -0.16 | 0.11 | 0.85 (0.68, 1.06) | 0.217 |
|  | ChristensenellaceaeR.7group.id.11283 | 11 | Inverse variance weighted | -0.15 | 0.08 | 0.86 (0.73, 1.02) | 0.077 |
|  |  |  | MR Egger | -0.07 | 0.26 | 0.94 (0.56, 1.55) | 0.801 |
|  |  |  | Simple mode | -0.35 | 0.20 | 0.70 (0.47, 1.05) | 0.115 |
|  |  |  | Weighted median | -0.11 | 0.17 | 0.89 (0.71, 1.12) | 0.330 |
|  |  |  | Weighted mode | -0.34 | 0.19 | 0.71 (0.49, 1.03) | 0.102 |
|  | Clostridiumsensustricto1.id.1873 | 9 | Inverse variance weighted | -0.00 | 0.11 | 1.00 (0.81, 1.23) | 0.994 |
|  |  |  | MR Egger | -0.16 | 0.24 | 0.85 (0.53, 1.37) | 0.526 |
|  |  |  | Simple mode | -0.03 | 0.16 | 0.97 (0.71, 1.34) | 0.877 |
|  |  |  | Weighted median | -0.03 | 0.10 | 0.97 (0.80, 1.19) | 0.796 |
|  |  |  | Weighted mode | -0.03 | 0.13 | 0.97 (0.75, 1.26) | 0.846 |
|  | Collinsella.id.815 | 12 | Inverse variance weighted | -0.03 | 0.10 | 0.97 (0.80, 1.17) | 0.730 |
|  |  |  | MR Egger | -0.44 | 0.38 | 0.64 (0.30, 1.37) | 0.280 |
|  |  |  | Simple mode | -0.11 | 0.22 | 0.89 (0.58, 1.38) | 0.622 |
|  |  |  | Weighted median | -0.04 | 0.11 | 0.96 (0.77, 1.19) | 0.701 |
|  |  |  | Weighted mode | -0.06 | 0.20 | 0.94 (0.64, 1.38) | 0.753 |
|  | Coprobacter.id.949 | 14 | Inverse variance weighted | 0.07 | 0.04 | 1.07 (0.98, 1.17) | 0.123 |
|  |  |  | MR Egger | 0.11 | 0.13 | 1.11 (0.86, 1.44) | 0.426 |
|  |  |  | Simple mode | 0.14 | 0.11 | 1.16 (0.94, 1.42) | 0.195 |
|  |  |  | Weighted median | 0.13 | 0.06 | 1.13 (1.01, 1.27) | 0.034 |
|  |  |  | Weighted mode | 0.14 | 0.09 | 1.15 (0.96, 1.38) | 0.159 |
|  | Coprococcus1.id.11301 | 14 | Inverse variance weighted | -0.02 | 0.08 | 0.98 (0.85, 1.15) | 0.843 |
|  |  |  | MR Egger | -0.33 | 0.17 | 0.72 (0.51, 1.00) | 0.071 |
|  |  |  | Simple mode | -0.13 | 0.16 | 0.88 (0.64, 1.21) | 0.451 |
|  |  |  | Weighted median | -0.12 | 0.10 | 0.89 (0.74, 1.07) | 0.223 |
|  |  |  | Weighted mode | -0.16 | 0.13 | 0.86 (0.67, 1.10) | 0.238 |
|  | Coprococcus2.id.11302 | 10 | Inverse variance weighted | 0.12 | 0.09 | 1.13 (0.95, 1.36) | 0.172 |
|  |  |  | MR Egger | 1.05 | 0.44 | 2.87 (1.20, 6.86) | 0.045 |
|  |  |  | Simple mode | -0.03 | 0.16 | 0.97 (0.71, 1.32) | 0.852 |
|  |  |  | Weighted median | 0.06 | 0.10 | 1.06 (0.87, 1.30) | 0.555 |
|  |  |  | Weighted mode | -0.01 | 0.16 | 0.99 (0.72, 1.35) | 0.933 |
|  | Coprococcus3.id.11303 | 10 | Inverse variance weighted | 0.12 | 0.09 | 1.12 (0.94, 1.34) | 0.190 |
|  |  |  | MR Egger | 0.38 | 0.37 | 1.46 (0.71, 3.00) | 0.328 |
|  |  |  | Simple mode | 0.27 | 0.21 | 1.31 (0.87, 1.99) | 0.231 |
|  |  |  | Weighted median | 0.20 | 0.12 | 1.22 (0.97, 1.54) | 0.086 |
|  |  |  | Weighted mode | 0.30 | 0.19 | 1.35 (0.93, 1.97) | 0.151 |
|  | DefluviitaleaceaeUCG011.id.11287 | 9 | Inverse variance weighted | 0.01 | 0.06 | 1.01 (0.90, 1.14) | 0.881 |
|  |  |  | MR Egger | -0.02 | 0.21 | 0.98 (0.65, 1.46) | 0.913 |
|  |  |  | Simple mode | -0.03 | 0.12 | 0.97 (0.77, 1.22) | 0.787 |
|  |  |  | Weighted median | 0.00 | 0.08 | 1.00 (0.87, 1.16) | 0.955 |
|  |  |  | Weighted mode | -0.04 | 0.11 | 0.96 (0.77, 1.20) | 0.737 |
|  | Desulfovibrio.id.3173 | 11 | Inverse variance weighted | 0.00 | 0.06 | 1.00 (0.89, 1.13) | 0.962 |
|  |  |  | MR Egger | -0.10 | 0.18 | 0.90 (0.63, 1.29) | 0.591 |
|  |  |  | Simple mode | -0.04 | 0.11 | 0.96 (0.77, 1.20) | 0.719 |
|  |  |  | Weighted median | -0.04 | 0.08 | 0.96 (0.82, 1.12) | 0.606 |
|  |  |  | Weighted mode | -0.04 | 0.11 | 0.96 (0.77, 1.19) | 0.711 |
|  | Dialister.id.2183 | 12 | Inverse variance weighted | -0.02 | 0.09 | 0.98 (0.82, 1.16) | 0.784 |
|  |  |  | MR Egger | -0.20 | 0.38 | 0.82 (0.39, 1.73) | 0.611 |
|  |  |  | Simple mode | -0.08 | 0.17 | 0.92 (0.67, 1.28) | 0.643 |
|  |  |  | Weighted median | -0.07 | 0.10 | 0.93 (0.77, 1.13) | 0.455 |
|  |  |  | Weighted mode | -0.08 | 0.16 | 0.92 (0.68, 1.26) | 0.629 |
|  | Dorea.id.1997 | 11 | Inverse variance weighted | -0.05 | 0.08 | 0.95 (0.81, 1.11) | 0.509 |
|  |  |  | MR Egger | 0.03 | 0.23 | 1.03 (0.65, 1.63) | 0.902 |
|  |  |  | Simple mode | -0.12 | 0.18 | 0.89 (0.62, 1.26) | 0.509 |
|  |  |  | Weighted median | -0.08 | 0.11 | 0.93 (0.75, 1.14) | 0.472 |
|  |  |  | Weighted mode | -0.08 | 0.16 | 0.92 (0.67, 1.27) | 0.630 |
|  | Eggerthella.id.819 | 10 | Inverse variance weighted | 0.03 | 0.06 | 1.03 (0.91, 1.16) | 0.650 |
|  |  |  | MR Egger | 0.07 | 0.30 | 1.07 (0.60, 1.93) | 0.817 |
|  |  |  | Simple mode | 0.13 | 0.10 | 1.13 (0.94, 1.38) | 0.234 |
|  |  |  | Weighted median | 0.10 | 0.07 | 1.11 (0.97, 1.27) | 0.146 |
|  |  |  | Weighted mode | 0.13 | 0.10 | 1.14 (0.94, 1.37) | 0.220 |
|  | Eisenbergiella.id.11304 | 12 | Inverse variance weighted | -0.03 | 0.05 | 0.98 (0.89, 1.07) | 0.599 |
|  |  |  | MR Egger | 0.18 | 0.37 | 1.19 (0.58, 2.45) | 0.641 |
|  |  |  | Simple mode | -0.11 | 0.13 | 0.89 (0.70, 1.14) | 0.385 |
|  |  |  | Weighted median | -0.02 | 0.06 | 0.98 (0.86, 1.10) | 0.704 |
|  |  |  | Weighted mode | -0.11 | 0.13 | 0.89 (0.70, 1.14) | 0.390 |
|  | Enterorhabdus.id.820 | 10 | Inverse variance weighted | 0.04 | 0.06 | 1.05 (0.94, 1.17) | 0.421 |
|  |  |  | MR Egger | -0.05 | 0.17 | 0.95 (0.68, 1.32) | 0.768 |
|  |  |  | Simple mode | 0.14 | 0.14 | 1.15 (0.87, 1.52) | 0.348 |
|  |  |  | Weighted median | 0.10 | 0.08 | 1.11 (0.95, 1.29) | 0.192 |
|  |  |  | Weighted mode | 0.15 | 0.14 | 1.16 (0.87, 1.53) | 0.338 |
|  | Erysipelatoclostridium.id.11381 | 16 | Inverse variance weighted | 0.01 | 0.05 | 1.01 (0.91, 1.12) | 0.828 |
|  |  |  | MR Egger | 0.24 | 0.21 | 1.27 (0.85, 1.91) | 0.266 |
|  |  |  | Simple mode | 0.07 | 0.12 | 1.07 (0.84, 1.36) | 0.601 |
|  |  |  | Weighted median | 0.05 | 0.07 | 1.05 (0.91, 1.21) | 0.503 |
|  |  |  | Weighted mode | 0.05 | 0.13 | 1.06 (0.82, 1.36) | 0.685 |
|  | ErysipelotrichaceaeUCG003.id.11384 | 17 | Inverse variance weighted | 0.03 | 0.07 | 1.03 (0.89, 1.19) | 0.682 |
|  |  |  | MR Egger | 0.09 | 0.21 | 1.09 (0.73, 1.65) | 0.674 |
|  |  |  | Simple mode | 0.15 | 0.16 | 1.16 (0.85, 1.58) | 0.357 |
|  |  |  | Weighted median | 0.13 | 0.09 | 1.14 (0.96, 1.35) | 0.133 |
|  |  |  | Weighted mode | 0.16 | 0.16 | 1.17 (0.86, 1.61) | 0.334 |
|  | Escherichia.Shigella.id.3504 | 15 | Inverse variance weighted | 0.04 | 0.06 | 1.04 (0.92, 1.17) | 0.531 |
|  |  |  | MR Egger | 0.06 | 0.17 | 1.06 (0.76, 1.49) | 0.723 |
|  |  |  | Simple mode | -0.07 | 0.16 | 0.93 (0.68, 1.26) | 0.640 |
|  |  |  | Weighted median | -0.00 | 0.08 | 1.00 (0.85, 1.17) | 0.987 |
|  |  |  | Weighted mode | -0.07 | 0.15 | 0.93 (0.70, 1.24) | 0.632 |
|  | Faecalibacterium.id.2057 | 13 | Inverse variance weighted | -0.05 | 0.07 | 0.95 (0.83, 1.08) | 0.418 |
|  |  |  | MR Egger | -0.13 | 0.14 | 0.87 (0.67, 1.14) | 0.348 |
|  |  |  | Simple mode | 0.01 | 0.15 | 1.01 (0.76, 1.35) | 0.929 |
|  |  |  | Weighted median | -0.00 | 0.09 | 1.00 (0.84, 1.19) | 0.992 |
|  |  |  | Weighted mode | 0.03 | 0.13 | 1.03 (0.80, 1.33) | 0.821 |
|  | FamilyXIIIAD3011group.id.11293 | 14 | Inverse variance weighted | -0.11 | 0.08 | 0.90 (0.76, 1.06) | 0.192 |
|  |  |  | MR Egger | -0.04 | 0.41 | 0.96 (0.43, 2.15) | 0.918 |
|  |  |  | Simple mode | 0.01 | 0.19 | 1.01 (0.70, 1.46) | 0.949 |
|  |  |  | Weighted median | -0.09 | 0.10 | 0.92 (0.75, 1.12) | 0.397 |
|  |  |  | Weighted mode | -0.00 | 0.17 | 1.00 (0.71, 1.40) | 0.999 |
|  | FamilyXIIIUCG001.id.11294 | 10 | Inverse variance weighted | -0.08 | 0.07 | 0.92 (0.80, 1.06) | 0.245 |
|  |  |  | MR Egger | 0.07 | 0.21 | 1.07 (0.71, 1.62) | 0.742 |
|  |  |  | Simple mode | -0.07 | 0.14 | 0.94 (0.70, 1.24) | 0.655 |
|  |  |  | Weighted median | -0.09 | 0.10 | 0.92 (0.76, 1.11) | 0.370 |
|  |  |  | Weighted mode | -0.08 | 0.14 | 0.93 (0.71, 1.21) | 0.588 |
|  | Flavonifractor.id.2059 | 8 | Inverse variance weighted | 0.03 | 0.08 | 1.03 (0.88, 1.22) | 0.680 |
|  |  |  | MR Egger | -0.32 | 0.31 | 0.72 (0.39, 1.34) | 0.343 |
|  |  |  | Simple mode | 0.03 | 0.16 | 1.03 (0.74, 1.41) | 0.882 |
|  |  |  | Weighted median | 0.04 | 0.10 | 1.04 (0.85, 1.26) | 0.708 |
|  |  |  | Weighted mode | 0.04 | 0.16 | 1.04 (0.76, 1.43) | 0.826 |
|  | Fusicatenibacter.id.11305 | 19 | Inverse variance weighted | -0.13 | 0.06 | 0.88 (0.77, 1.00) | 0.046 |
|  |  |  | MR Egger | 0.04 | 0.26 | 1.04 (0.63, 1.72) | 0.878 |
|  |  |  | Simple mode | 0.01 | 0.18 | 1.01 (0.71, 1.44) | 0.955 |
|  |  |  | Weighted median | -0.08 | 0.09 | 0.92 (0.78, 1.09) | 0.327 |
|  |  |  | Weighted mode | 0.05 | 0.18 | 1.05 (0.74, 1.50) | 0.784 |
|  | Gordonibacter.id.821 | 14 | Inverse variance weighted | 0.01 | 0.04 | 1.01 (0.94, 1.08) | 0.779 |
|  |  |  | MR Egger | -0.08 | 0.14 | 0.92 (0.70, 1.21) | 0.576 |
|  |  |  | Simple mode | -0.03 | 0.08 | 0.97 (0.82, 1.13) | 0.690 |
|  |  |  | Weighted median | -0.00 | 0.05 | 1.00 (0.91, 1.10) | 0.965 |
|  |  |  | Weighted mode | -0.02 | 0.07 | 0.98 (0.84, 1.13) | 0.747 |
|  | Haemophilus.id.3698 | 14 | Inverse variance weighted | 0.05 | 0.07 | 1.05 (0.92, 1.20) | 0.436 |
|  |  |  | MR Egger | -0.13 | 0.17 | 0.88 (0.64, 1.22) | 0.465 |
|  |  |  | Simple mode | 0.14 | 0.13 | 1.15 (0.89, 1.49) | 0.301 |
|  |  |  | Weighted median | 0.04 | 0.07 | 1.04 (0.90, 1.21) | 0.588 |
|  |  |  | Weighted mode | 0.04 | 0.11 | 1.05 (0.85, 1.29) | 0.690 |
|  | Holdemanella.id.11393 | 14 | Inverse variance weighted | 0.03 | 0.05 | 1.03 (0.94, 1.13) | 0.499 |
|  |  |  | MR Egger | -0.12 | 0.15 | 0.89 (0.66, 1.19) | 0.433 |
|  |  |  | Simple mode | 0.02 | 0.10 | 1.02 (0.84, 1.25) | 0.829 |
|  |  |  | Weighted median | 0.02 | 0.06 | 1.02 (0.90, 1.15) | 0.803 |
|  |  |  | Weighted mode | 0.02 | 0.10 | 1.02 (0.85, 1.23) | 0.831 |
|  | Holdemania.id.2157 | 18 | Inverse variance weighted | 0.06 | 0.05 | 1.06 (0.97, 1.16) | 0.186 |
|  |  |  | MR Egger | -0.02 | 0.13 | 0.98 (0.76, 1.28) | 0.910 |
|  |  |  | Simple mode | 0.07 | 0.10 | 1.07 (0.88, 1.30) | 0.512 |
|  |  |  | Weighted median | 0.06 | 0.06 | 1.06 (0.94, 1.19) | 0.367 |
|  |  |  | Weighted mode | 0.07 | 0.09 | 1.08 (0.90, 1.29) | 0.448 |
|  | Howardella.id.2000 | 10 | Inverse variance weighted | 0.01 | 0.04 | 1.01 (0.93, 1.10) | 0.760 |
|  |  |  | MR Egger | -0.05 | 0.19 | 0.95 (0.65, 1.39) | 0.805 |
|  |  |  | Simple mode | 0.00 | 0.09 | 1.00 (0.84, 1.19) | 0.991 |
|  |  |  | Weighted median | 0.01 | 0.06 | 1.01 (0.90, 1.12) | 0.927 |
|  |  |  | Weighted mode | 0.00 | 0.08 | 1.00 (0.85, 1.18) | 0.968 |
|  | Hungatella.id.11306 | 5 | Inverse variance weighted | -0.18 | 0.08 | 0.84 (0.71, 0.98) | 0.026 |
|  |  |  | MR Egger | -0.27 | 0.59 | 0.76 (0.24, 2.41) | 0.675 |
|  |  |  | Simple mode | -0.19 | 0.14 | 0.82 (0.63, 1.08) | 0.227 |
|  |  |  | Weighted median | -0.16 | 0.09 | 0.85 (0.71, 1.02) | 0.081 |
|  |  |  | Weighted mode | -0.11 | 0.13 | 0.89 (0.69, 1.15) | 0.440 |
|  | Intestinibacter.id.11345 | 14 | Inverse variance weighted | -0.07 | 0.06 | 0.93 (0.82, 1.05) | 0.232 |
|  |  |  | MR Egger | -0.16 | 0.20 | 0.86 (0.57, 1.27) | 0.458 |
|  |  |  | Simple mode | -0.07 | 0.16 | 0.94 (0.69, 1.28) | 0.682 |
|  |  |  | Weighted median | -0.09 | 0.08 | 0.92 (0.78, 1.07) | 0.276 |
|  |  |  | Weighted mode | -0.08 | 0.15 | 0.93 (0.69, 1.24) | 0.620 |
|  | Intestinimonas.id.2062 | 20 | Inverse variance weighted | 0.02 | 0.06 | 1.02 (0.91, 1.15) | 0.683 |
|  |  |  | MR Egger | -0.14 | 0.15 | 0.87 (0.65, 1.16) | 0.341 |
|  |  |  | Simple mode | -0.04 | 0.13 | 0.96 (0.74, 1.25) | 0.784 |
|  |  |  | Weighted median | -0.03 | 0.07 | 0.98 (0.85, 1.12) | 0.723 |
|  |  |  | Weighted mode | -0.04 | 0.14 | 0.96 (0.73, 1.27) | 0.796 |
|  | Lachnoclostridium.id.11308 | 14 | Inverse variance weighted | -0.06 | 0.08 | 0.94 (0.80, 1.10) | 0.423 |
|  |  |  | MR Egger | -0.13 | 0.28 | 0.88 (0.51, 1.53) | 0.664 |
|  |  |  | Simple mode | -0.10 | 0.17 | 0.90 (0.65, 1.25) | 0.550 |
|  |  |  | Weighted median | -0.09 | 0.11 | 0.91 (0.74, 1.12) | 0.385 |
|  |  |  | Weighted mode | -0.10 | 0.17 | 0.91 (0.65, 1.27) | 0.584 |
|  | Lachnospira.id.2004 | 7 | Inverse variance weighted | -0.09 | 0.17 | 0.92 (0.65, 1.29) | 0.616 |
|  |  |  | MR Egger | 0.44 | 1.04 | 1.56 (0.20, 11.95) | 0.689 |
|  |  |  | Simple mode | 0.29 | 0.25 | 1.33 (0.82, 2.17) | 0.296 |
|  |  |  | Weighted median | 0.16 | 0.17 | 1.17 (0.84, 1.63) | 0.360 |
|  |  |  | Weighted mode | 0.29 | 0.27 | 1.33 (0.79, 2.25) | 0.328 |
|  | LachnospiraceaeFCS020group.id.11314 | 16 | Inverse variance weighted | -0.01 | 0.07 | 0.99 (0.87, 1.13) | 0.911 |
|  |  |  | MR Egger | -0.02 | 0.17 | 0.99 (0.71, 1.37) | 0.931 |
|  |  |  | Simple mode | -0.22 | 0.18 | 0.80 (0.57, 1.14) | 0.238 |
|  |  |  | Weighted median | 0.03 | 0.08 | 1.03 (0.88, 1.22) | 0.685 |
|  |  |  | Weighted mode | 0.11 | 0.15 | 1.12 (0.83, 1.50) | 0.460 |
|  | LachnospiraceaeNC2004group.id.11316 | 10 | Inverse variance weighted | 0.10 | 0.05 | 1.11 (0.99, 1.23) | 0.063 |
|  |  |  | MR Egger | 0.06 | 0.23 | 1.06 (0.67, 1.66) | 0.811 |
|  |  |  | Simple mode | 0.04 | 0.11 | 1.04 (0.84, 1.29) | 0.728 |
|  |  |  | Weighted median | 0.07 | 0.07 | 1.07 (0.94, 1.22) | 0.322 |
|  |  |  | Weighted mode | 0.04 | 0.11 | 1.04 (0.84, 1.28) | 0.731 |
|  | LachnospiraceaeND3007group.id.11317 | 3 | Inverse variance weighted | 0.33 | 0.16 | 1.39 (1.02, 1.89) | 0.038 |
|  |  |  | MR Egger | -0.83 | 2.65 | 0.44 (0.00, 78.11) | 0.806 |
|  |  |  | Simple mode | 0.23 | 0.22 | 1.26 (0.82, 1.93) | 0.399 |
|  |  |  | Weighted median | 0.26 | 0.19 | 1.30 (0.89, 1.91) | 0.175 |
|  |  |  | Weighted mode | 0.24 | 0.23 | 1.27 (0.81, 1.98) | 0.404 |
|  | LachnospiraceaeNK4A136group.id.11319 | 16 | Inverse variance weighted | -0.08 | 0.06 | 0.93 (0.82, 1.04) | 0.199 |
|  |  |  | MR Egger | 0.09 | 0.13 | 1.09 (0.85, 1.40) | 0.511 |
|  |  |  | Simple mode | -0.05 | 0.14 | 0.96 (0.72, 1.27) | 0.757 |
|  |  |  | Weighted median | -0.04 | 0.08 | 0.96 (0.82, 1.12) | 0.599 |
|  |  |  | Weighted mode | -0.05 | 0.10 | 0.96 (0.79, 1.16) | 0.648 |
|  | LachnospiraceaeUCG001.id.11321 | 16 | Inverse variance weighted | 0.02 | 0.05 | 1.02 (0.92, 1.14) | 0.675 |
|  |  |  | MR Egger | 0.07 | 0.25 | 1.07 (0.66, 1.74) | 0.797 |
|  |  |  | Simple mode | -0.08 | 0.13 | 0.93 (0.72, 1.19) | 0.561 |
|  |  |  | Weighted median | -0.02 | 0.07 | 0.98 (0.85, 1.13) | 0.820 |
|  |  |  | Weighted mode | 0.09 | 0.13 | 1.09 (0.85, 1.40) | 0.504 |
|  | LachnospiraceaeUCG004.id.11324 | 13 | Inverse variance weighted | -0.12 | 0.07 | 0.89 (0.77, 1.02) | 0.099 |
|  |  |  | MR Egger | -0.29 | 0.27 | 0.75 (0.46, 1.26) | 0.298 |
|  |  |  | Simple mode | -0.09 | 0.19 | 0.91 (0.64, 1.32) | 0.639 |
|  |  |  | Weighted median | -0.10 | 0.10 | 0.90 (0.74, 1.10) | 0.304 |
|  |  |  | Weighted mode | -0.10 | 0.17 | 0.91 (0.65, 1.27) | 0.584 |
|  | LachnospiraceaeUCG008.id.11328 | 12 | Inverse variance weighted | -0.05 | 0.05 | 0.95 (0.86, 1.05) | 0.292 |
|  |  |  | MR Egger | 0.28 | 0.26 | 1.32 (0.80, 2.18) | 0.307 |
|  |  |  | Simple mode | -0.03 | 0.11 | 0.97 (0.78, 1.21) | 0.811 |
|  |  |  | Weighted median | -0.03 | 0.07 | 0.97 (0.85, 1.11) | 0.630 |
|  |  |  | Weighted mode | -0.02 | 0.12 | 0.98 (0.77, 1.25) | 0.853 |
|  | LachnospiraceaeUCG010.id.11330 | 11 | Inverse variance weighted | 0.03 | 0.07 | 1.03 (0.90, 1.18) | 0.675 |
|  |  |  | MR Egger | -0.06 | 0.19 | 0.94 (0.65, 1.36) | 0.751 |
|  |  |  | Simple mode | -0.05 | 0.13 | 0.95 (0.74, 1.23) | 0.729 |
|  |  |  | Weighted median | -0.03 | 0.09 | 0.97 (0.80, 1.16) | 0.711 |
|  |  |  | Weighted mode | -0.04 | 0.12 | 0.96 (0.76, 1.23) | 0.764 |
|  | Lactobacillus.id.1837 | 9 | Inverse variance weighted | -0.08 | 0.06 | 0.92 (0.83, 1.03) | 0.160 |
|  |  |  | MR Egger | -0.18 | 0.16 | 0.83 (0.61, 1.14) | 0.287 |
|  |  |  | Simple mode | -0.10 | 0.10 | 0.90 (0.74, 1.10) | 0.338 |
|  |  |  | Weighted median | -0.10 | 0.07 | 0.91 (0.80, 1.04) | 0.169 |
|  |  |  | Weighted mode | -0.11 | 0.10 | 0.89 (0.73, 1.09) | 0.303 |
|  | Lactococcus.id.1851 | 11 | Inverse variance weighted | 0.02 | 0.04 | 1.02 (0.93, 1.11) | 0.720 |
|  |  |  | MR Egger | -0.03 | 0.23 | 0.97 (0.61, 1.54) | 0.906 |
|  |  |  | Simple mode | -0.00 | 0.09 | 1.00 (0.83, 1.20) | 0.961 |
|  |  |  | Weighted median | 0.04 | 0.06 | 1.04 (0.93, 1.16) | 0.502 |
|  |  |  | Weighted mode | 0.01 | 0.09 | 1.01 (0.85, 1.20) | 0.905 |
|  | Marvinbryantia.id.2005 | 12 | Inverse variance weighted | -0.04 | 0.07 | 0.96 (0.84, 1.10) | 0.559 |
|  |  |  | MR Egger | 0.34 | 0.25 | 1.40 (0.85, 2.31) | 0.212 |
|  |  |  | Simple mode | -0.15 | 0.15 | 0.86 (0.65, 1.15) | 0.334 |
|  |  |  | Weighted median | -0.07 | 0.09 | 0.93 (0.78, 1.10) | 0.404 |
|  |  |  | Weighted mode | -0.14 | 0.15 | 0.87 (0.64, 1.17) | 0.377 |
|  | Methanobrevibacter.id.123 | 8 | Inverse variance weighted | 0.01 | 0.05 | 1.00 (0.92, 1.10) | 0.911 |
|  |  |  | MR Egger | 0.02 | 0.17 | 1.01 (0.72, 1.43) | 0.933 |
|  |  |  | Simple mode | -0.03 | 0.09 | 0.97 (0.81, 1.17) | 0.786 |
|  |  |  | Weighted median | -0.01 | 0.06 | 0.99 (0.89, 1.11) | 0.928 |
|  |  |  | Weighted mode | -0.03 | 0.09 | 0.97 (0.81, 1.17) | 0.772 |
|  | Odoribacter.id.952 | 9 | Inverse variance weighted | -0.06 | 0.09 | 0.94 (0.80, 1.11) | 0.471 |
|  |  |  | MR Egger | 0.07 | 0.29 | 1.07 (0.60, 1.90) | 0.829 |
|  |  |  | Simple mode | 0.09 | 0.20 | 1.10 (0.74, 1.62) | 0.660 |
|  |  |  | Weighted median | -0.01 | 0.12 | 0.99 (0.78, 1.26) | 0.942 |
|  |  |  | Weighted mode | 0.09 | 0.19 | 1.10 (0.76, 1.59) | 0.642 |
|  | Olsenella.id.822 | 11 | Inverse variance weighted | 0.16 | 0.05 | 1.02 (0.92, 1.13) | 0.768 |
|  |  |  | MR Egger | -0.33 | 0.17 | 0.72 (0.51, 1.01) | 0.088 |
|  |  |  | Simple mode | 0.03 | 0.10 | 1.03 (0.85, 1.26) | 0.750 |
|  |  |  | Weighted median | 0.03 | 0.06 | 1.03 (0.92, 1.15) | 0.596 |
|  |  |  | Weighted mode | 0.03 | 0.10 | 1.03 (0.86, 1.25) | 0.749 |
|  | Oscillibacter.id.2063 | 16 | Inverse variance weighted | 0.07 | 0.05 | 1.07 (0.97, 1.18) | 0.170 |
|  |  |  | MR Egger | 0.22 | 0.19 | 1.25 (0.86, 1.80) | 0.257 |
|  |  |  | Simple mode | 0.08 | 0.11 | 1.09 (0.87, 1.36) | 0.483 |
|  |  |  | Weighted median | 0.06 | 0.06 | 1.06 (0.94, 1.20) | 0.362 |
|  |  |  | Weighted mode | 0.06 | 0.10 | 1.07 (0.87, 1.31) | 0.547 |
|  | Oscillospira.id.2064 | 9 | Inverse variance weighted | 0.07 | 0.07 | 1.07 (0.93, 1.23) | 0.338 |
|  |  |  | MR Egger | 0.07 | 0.30 | 1.08 (0.60, 1.93) | 0.814 |
|  |  |  | Simple mode | 0.03 | 0.13 | 1.03 (0.79, 1.34) | 0.837 |
|  |  |  | Weighted median | 0.05 | 0.09 | 1.05 (0.88, 1.25) | 0.614 |
|  |  |  | Weighted mode | 0.05 | 0.13 | 1.05 (0.82, 1.34) | 0.728 |
|  | Oxalobacter.id.2978 | 12 | Inverse variance weighted | -0.01 | 0.04 | 0.99 (0.92, 1.08) | 0.877 |
|  |  |  | MR Egger | -0.08 | 0.18 | 0.92 (0.65, 1.30) | 0.659 |
|  |  |  | Simple mode | 0.08 | 0.09 | 1.08 (0.90, 1.30) | 0.412 |
|  |  |  | Weighted median | -0.01 | 0.05 | 0.99 (0.89, 1.10) | 0.885 |
|  |  |  | Weighted mode | 0.06 | 0.09 | 1.06 (0.89, 1.28) | 0.516 |
|  | Parabacteroides.id.954 | 10 | Inverse variance weighted | -0.03 | 0.08 | 0.97 (0.83, 1.14) | 0.740 |
|  |  |  | MR Egger | -0.21 | 0.22 | 0.81 (0.53, 1.23) | 0.350 |
|  |  |  | Simple mode | -0.13 | 0.17 | 0.88 (0.63, 1.23) | 0.477 |
|  |  |  | Weighted median | -0.12 | 0.10 | 0.89 (0.72, 1.09) | 0.253 |
|  |  |  | Weighted mode | -0.14 | 0.15 | 0.87 (0.65, 1.18) | 0.403 |
|  | Paraprevotella.id.962 | 13 | Inverse variance weighted | -0.00 | 0.05 | 1.00 (0.90, 1.11) | 0.993 |
|  |  |  | MR Egger | 0.05 | 0.18 | 1.05 (0.74, 1.49) | 0.795 |
|  |  |  | Simple mode | -0.09 | 0.11 | 0.92 (0.74, 1.14) | 0.452 |
|  |  |  | Weighted median | -0.06 | 0.06 | 0.94 (0.83, 1.07) | 0.379 |
|  |  |  | Weighted mode | -0.09 | 0.10 | 0.91 (0.75, 1.11) | 0.369 |
|  | Parasutterella.id.2892 | 17 | Inverse variance weighted | -0.01 | 0.06 | 0.99 (0.88, 1.11) | 0.838 |
|  |  |  | MR Egger | 0.17 | 0.20 | 1.19 (0.81, 1.74) | 0.397 |
|  |  |  | Simple mode | 0.17 | 0.15 | 1.19 (0.88, 1.60) | 0.272 |
|  |  |  | Weighted median | -0.03 | 0.08 | 0.97 (0.83, 1.13) | 0.688 |
|  |  |  | Weighted mode | 0.05 | 0.13 | 1.05 (0.82, 1.34) | 0.726 |
|  | Peptococcus.id.2037 | 16 | Inverse variance weighted | 0.01 | 0.04 | 1.01 (0.93, 1.09) | 0.825 |
|  |  |  | MR Egger | -0.03 | 0.15 | 0.97 (0.73, 1.29) | 0.829 |
|  |  |  | Simple mode | 0.05 | 0.09 | 1.05 (0.88, 1.25) | 0.603 |
|  |  |  | Weighted median | 0.03 | 0.05 | 1.03 (0.93, 1.14) | 0.566 |
|  |  |  | Weighted mode | 0.05 | 0.09 | 1.05 (0.88, 1.25) | 0.572 |
|  | Phascolarctobacterium.id.2168 | 12 | Inverse variance weighted | -0.09 | 0.07 | 0.92 (0.81, 1.05) | 0.196 |
|  |  |  | MR Egger | 0.35 | 0.24 | 1.42 (0.89, 2.28) | 0.177 |
|  |  |  | Simple mode | 0.02 | 0.18 | 1.02 (0.72, 1.44) | 0.924 |
|  |  |  | Weighted median | -0.04 | 0.09 | 0.96 (0.80, 1.14) | 0.625 |
|  |  |  | Weighted mode | 0.01 | 0.16 | 1.01 (0.74, 1.37) | 0.957 |
|  | Prevotella7.id.11182 | 11 | Inverse variance weighted | -0.06 | 0.04 | 0.94 (0.87, 1.02) | 0.128 |
|  |  |  | MR Egger | -0.31 | 0.24 | 0.73 (0.46, 1.16) | 0.218 |
|  |  |  | Simple mode | -0.14 | 0.10 | 0.87 (0.72, 1.05) | 0.184 |
|  |  |  | Weighted median | -0.05 | 0.05 | 0.95 (0.85, 1.06) | 0.337 |
|  |  |  | Weighted mode | -0.02 | 0.10 | 1.02 (0.84, 1.24) | 0.853 |
|  | Prevotella9.id.11183 | 19 | Inverse variance weighted | 0.03 | 0.05 | 1.03 (0.94, 1.13) | 0.513 |
|  |  |  | MR Egger | -0.04 | 0.13 | 0.96 (0.75, 1.23) | 0.730 |
|  |  |  | Simple mode | 0.13 | 0.12 | 1.13 (0.89, 1.44) | 0.315 |
|  |  |  | Weighted median | 0.06 | 0.06 | 1.07 (0.95, 1.20) | 0.298 |
|  |  |  | Weighted mode | 0.14 | 0.12 | 1.15 (0.91, 1.45) | 0.270 |
|  | RikenellaceaeRC9gutgroup.id.11191 | 13 | Inverse variance weighted | -0.07 | 0.04 | 0.93 (0.86, 1.02) | 0.109 |
|  |  |  | MR Egger | -0.22 | 0.27 | 0.81 (0.47, 1.38) | 0.448 |
|  |  |  | Simple mode | -0.03 | 0.09 | 0.97 (0.81, 0.17) | 0.793 |
|  |  |  | Weighted median | -0.05 | 0.05 | 0.95 (0.86, 1.05) | 0.277 |
|  |  |  | Weighted mode | -0.03 | 0.09 | 0.97 (0.81, 1.16) | 0.753 |
|  | Romboutsia.id.11347 | 14 | Inverse variance weighted | 0.01 | 0.06 | 1.01 (0.90, 1.14) | 0.825 |
|  |  |  | MR Egger | -0.14 | 0.16 | 0.87 (0.64, 1.18) | 0.385 |
|  |  |  | Simple mode | 0.02 | 0.12 | 1.02 (0.80, 1.30) | 0.869 |
|  |  |  | Weighted median | 0.02 | 0.08 | 1.02 (0.87, 1.19) | 0.836 |
|  |  |  | Weighted mode | -0.04 | 0.12 | 0.96 (0.75, 1.23) | 0.774 |
|  | Roseburia.id.2012 | 17 | Inverse variance weighted | -0.13 | 0.08 | 0.88 (0.76, 1.02) | 0.096 |
|  |  |  | MR Egger | -0.33 | 0.20 | 0.72 (0.48, 1.07) | 0.125 |
|  |  |  | Simple mode | -0.28 | 0.16 | 0.76 (0.55, 1.04) | 0.108 |
|  |  |  | Weighted median | -0.23 | 0.10 | 0.80 (0.66, 0.96) | 0.017 |
|  |  |  | Weighted mode | -0.30 | 0.15 | 0.74 (0.55, 1.00) | 0.066 |
|  | Ruminiclostridium5.id.11355 | 15 | Inverse variance weighted | -0.09 | 0.07 | 0.91 (0.79, 1.05) | 0.194 |
|  |  |  | MR Egger | -0.13 | 0.24 | 0.88 (0.56, 1.40) | 0.600 |
|  |  |  | Simple mode | -0.23 | 0.17 | 0.80 (0.57, 1.11) | 0.199 |
|  |  |  | Weighted median | -0.12 | 0.10 | 0.88 (0.73, 1.07) | 0.208 |
|  |  |  | Weighted mode | -0.20 | 0.15 | 0.82 (0.61, 1.11) | 0.225 |
|  | Ruminiclostridium6.id.11356 | 16 | Inverse variance weighted | 0.05 | 0.06 | 1.05 (0.93, 1.18) | 0.426 |
|  |  |  | MR Egger | -0.15 | 0.15 | 0.86 (0.65, 1.15) | 0.329 |
|  |  |  | Simple mode | 0.01 | 0.15 | 1.01 (0.75, 1.36) | 0.946 |
|  |  |  | Weighted median | 0.04 | 0.08 | 1.04 (0.89, 1.23) | 0.604 |
|  |  |  | Weighted mode | 0.02 | 0.13 | 1.02 (0.79, 1.32) | 0.864 |
|  | Ruminiclostridium9.id.11357 | 15 | Inverse variance weighted | 0.03 | 0.09 | 1.03 (0.87, 1.23) | 0.698 |
|  |  |  | MR Egger | 0.12 | 0.42 | 1.12 (0.49, 2.57) | 0.789 |
|  |  |  | Simple mode | -0.16 | 0.21 | 0.85 (0.56, 1.29) | 0.453 |
|  |  |  | Weighted median | -0.07 | 0.10 | 0.93 (0.76, 1.14) | 0.489 |
|  |  |  | Weighted mode | -0.16 | 0.22 | 0.85 (0.55, 1.30) | 0.462 |
|  | RuminococcaceaeNK4A214group.id.11358 | 14 | Inverse variance weighted | 0.02 | 0.08 | 1.02 (0.88, 1.18) | 0.773 |
|  |  |  | MR Egger | 0.10 | 0.22 | 1.11 (0.72, 1.70) | 0.651 |
|  |  |  | Simple mode | 0.12 | 0.16 | 1.13 (0.82, 1.56) | 0.463 |
|  |  |  | Weighted median | 0.04 | 0.09 | 1.04 (0.87, 1.25) | 0.650 |
|  |  |  | Weighted mode | 0.14 | 0.16 | 1.15 (0.85, 1.57) | 0.372 |
|  | RuminococcaceaeUCG002.id.11360 | 26 | Inverse variance weighted | 0.07 | 0.06 | 1.07 (0.95, 1.20) | 0.276 |
|  |  |  | MR Egger | -0.10 | 0.17 | 0.91 (0.65, 1.26) | 0.577 |
|  |  |  | Simple mode | 0.15 | 0.13 | 1.16 (0.89, 1.51) | 0.277 |
|  |  |  | Weighted median | 0.10 | 0.07 | 1.11 (0.96,1.28) | 0.164 |
|  |  |  | Weighted mode | 0.14 | 0.14 | 1.15 (0.87, 1.50) | 0.334 |
|  | RuminococcaceaeUCG003.id.11361 | 14 | Inverse variance weighted | 0.04 | 0.07 | 1.04 (0.91, 1.19) | 0.550 |
|  |  |  | MR Egger | -0.06 | 0.23 | 0.94 (0.60, 1.48) | 0.808 |
|  |  |  | Simple mode | -0.01 | 0.15 | 0.99 (0.75, 1.32) | 0.955 |
|  |  |  | Weighted median | 0.01 | 0.09 | 1.01 (0.84, 1.20) | 0.948 |
|  |  |  | Weighted mode | -0.01 | 0.14 | 0.99 (0.75, 1.30) | 0.942 |
|  | RuminococcaceaeUCG004.id.11362 | 11 | Inverse variance weighted | 0.03 | 0.06 | 1.04 (0.91, 1.17) | 0.584 |
|  |  |  | MR Egger | -0.47 | 0.35 | 0.63 (0.32, 1.24) | 0.212 |
|  |  |  | Simple mode | -0.01 | 0.13 | 0.99 (0.76, 1.28) | 0.941 |
|  |  |  | Weighted median | -0.00 | 0.08 | 1.00 (0.85, 1.17) | 0.956 |
|  |  |  | Weighted mode | -0.02 | 0.12 | 0.98 (0.78, 1.24) | 0.880 |
|  | RuminococcaceaeUCG005.id.11363 | 17 | Inverse variance weighted | 0.00 | 0.06 | 1.00 (0.89, 1.13) | 0.967 |
|  |  |  | MR Egger | -0.10 | 0.17 | 0.90 (0.65, 1.26) | 0.546 |
|  |  |  | Simple mode | 0.03 | 0.14 | 1.03 (0.79, 1.35) | 0.834 |
|  |  |  | Weighted median | 0.03 | 0.08 | 1.03 (0.87, 1.21) | 0.736 |
|  |  |  | Weighted mode | 0.05 | 0.13 | 1.05 (0.81, 1.35) | 0.714 |
|  | RuminococcaceaeUCG009.id.11366 | 13 | Inverse variance weighted | 0.06 | 0.06 | 1.06 (0.94, 1.19) | 0.357 |
|  |  |  | MR Egger | 0.22 | 0.26 | 1.25 (0.75, 2.10) | 0.412 |
|  |  |  | Simple mode | -0.09 | 0.13 | 0.92 (0.71, 1.18) | 0.512 |
|  |  |  | Weighted median | -0.00 | 0.08 | 1.00 (0.85, 1.16) | 0.976 |
|  |  |  | Weighted mode | -0.08 | 0.12 | 0.92 (0.72, 1.17) | 0.517 |
|  | RuminococcaceaeUCG010.id.11367 | 7 | Inverse variance weighted | 0.02 | 0.10 | 1.02 (0.84, 1.25) | 0.829 |
|  |  |  | MR Egger | 0.16 | 0.39 | 1.17 (0.54, 2.55) | 0.701 |
|  |  |  | Simple mode | -0.06 | 0.19 | 0.94 (0.65, 1.36) | 0.765 |
|  |  |  | Weighted median | 0.01 | 0.13 | 1.01 (0.79, 1.29) | 0.951 |
|  |  |  | Weighted mode | -0.03 | 0.19 | 0.97 (0.67, 1.41) | 0.880 |
|  | RuminococcaceaeUCG011.id.11368 | 7 | Inverse variance weighted | 0.10 | 0.05 | 1.11 (1.00, 1.23) | 0.063 |
|  |  |  | MR Egger | -0.25 | 0.24 | 0.78 (0.49, 1.24) | 0.335 |
|  |  |  | Simple mode | 0.21 | 0.12 | 1.24 (0.97, 1.58) | 0.134 |
|  |  |  | Weighted median | 0.09 | 0.06 | 1.10 (0.97, 1.24) | 0.144 |
|  |  |  | Weighted mode | -0.02 | 0.11 | 0.98 (0.77, 1.24) | 0.851 |
|  | RuminococcaceaeUCG013.id.11370 | 14 | Inverse variance weighted | -0.00 | 0.09 | 1.00 (0.83, 1.19) | 0.977 |
|  |  |  | MR Egger | 0.28 | 0.24 | 1.32 (0.82, 2.13) | 0.278 |
|  |  |  | Simple mode | 0.08 | 0.19 | 1.08 (0.75, 1.57) | 0.686 |
|  |  |  | Weighted median | 0.05 | 0.10 | 1.06 (0.87, 1.28) | 0.569 |
|  |  |  | Weighted mode | 0.12 | 0.15 | 1.13 (0.85, 1.50) | 0.426 |
|  | RuminococcaceaeUCG014.id.11371 | 18 | Inverse variance weighted | -0.03 | 0.06 | 0.97 (0.86, 1.09) | 0.597 |
|  |  |  | MR Egger | -0.13 | 0.18 | 0.88 (0.62, 1.25) | 0.479 |
|  |  |  | Simple mode | 0.08 | 0.17 | 1.08 (0.78, 1.51) | 0.642 |
|  |  |  | Weighted median | 0.02 | 0.08 | 1.02 (0.87, 1.20) | 0.794 |
|  |  |  | Weighted mode | 0.07 | 0.15 | 1.08 (0.80, 1.44) | 0.627 |
|  | Ruminococcus1.id.11373 | 14 | Inverse variance weighted | 0.03 | 0.08 | 1.03 (0.88, 1.20) | 0.742 |
|  |  |  | MR Egger | -0.29 | 0.21 | 0.75 (0.49,1.13) | 0.196 |
|  |  |  | Simple mode | -0.11 | 0.17 | 0.90 (0.64,1.26) | 0.549 |
|  |  |  | Weighted median | -0.00 | 0.10 | 1.00 (0.82,1.21) | 0.972 |
|  |  |  | Weighted mode | -0.11 | 0.17 | 0.89 (0.64,1.25) | 0.519 |
|  | Ruminococcus2.id.11374 | 15 | Inverse variance weighted | -0.09 | 0.07 | 0.92 (0.81, 1.04) | 0.191 |
|  |  |  | MR Egger | -0.06 | 0.18 | 0.94 (0.66, 1.33) | 0.734 |
|  |  |  | Simple mode | 0.09 | 0.16 | 1.09 (0.80, 1.48) | 0.583 |
|  |  |  | Weighted median | 0.05 | 0.09 | 1.05 (0.88, 1.25) | 0.604 |
|  |  |  | Weighted mode | 0.08 | 0.14 | 1.08 (0.82, 1.42) | 0.583 |
|  | Sellimonas.id.14369 | 11 | Inverse variance weighted | -0.00 | 0.04 | 1.00 (0.93, 1.07) | 0.958 |
|  |  |  | MR Egger | -0.40 | 0.22 | 0.67 (0.43, 1.03) | 0.099 |
|  |  |  | Simple mode | 0.00 | 0.09 | 1.00 (0.85, 1.19) | 0.973 |
|  |  |  | Weighted median | 0.01 | 0.05 | 1.01 (0.91, 1.10) | 0.914 |
|  |  |  | Weighted mode | 0.01 | 0.09 | 1.01 (0.85, 1.20) | 0.942 |
|  | Senegalimassilia.id.11160 | 7 | Inverse variance weighted | 0.04 | 0.08 | 1.04 (0.89, 1.21) | 0.627 |
|  |  |  | MR Egger | 0.22 | 0.34 | 1.25 (0.64, 2.44) | 0.544 |
|  |  |  | Simple mode | -0.01 | 0.15 | 0.99 (0.73, 1.33) | 0.937 |
|  |  |  | Weighted median | -0.01 | 0.09 | 0.99 (0.82, 1.19) | 0.932 |
|  |  |  | Weighted mode | 0.01 | 0.14 | 1.01 (0.77, 1.33) | 0.949 |
|  | Slackia.id.825 | 9 | Inverse variance weighted | -0.03 | 0.06 | 0.97 (0.87, 1.08) | 0.575 |
|  |  |  | MR Egger | 0.07 | 0.24 | 1.07 (0.67,1.70) | 0.792 |
|  |  |  | Simple mode | -0.08 | 0.12 | 0.92 (0.73, 1.16) | 0.504 |
|  |  |  | Weighted median | -0.05 | 0.07 | 0.96 (0.83, 1.11) | 0.540 |
|  |  |  | Weighted mode | -0.06 | 0.12 | 0.94 (0.75, 1.19) | 0.631 |
|  | Streptococcus.id.1853 | 16 | Inverse variance weighted | 0.12 | 0.08 | 1.13 (0.97, 1.32) | 0.117 |
|  |  |  | MR Egger | 0.38 | 0.31 | 1.46 (0.80, 2.66) | 0.234 |
|  |  |  | Simple mode | 0.04 | 0.15 | 1.05 (0.78, 1.40) | 0.769 |
|  |  |  | Weighted median | 0.07 | 0.09 | 1.07 (0.89, 1.28) | 0.467 |
|  |  |  | Weighted mode | 0.06 | 0.16 | 1.06 (0.78, 1.44) | 0.720 |
|  | Subdoligranulum.id.2070 | 14 | Inverse variance weighted | -0.00 | 0.09 | 1.00 (0.83, 1.20) | 0.974 |
|  |  |  | MR Egger | 0.25 | 0.26 | 1.28 (0.77, 2.15) | 0.360 |
|  |  |  | Simple mode | -0.14 | 0.18 | 0.87 (0.61, 1.23) | 0.440 |
|  |  |  | Weighted median | 0.02 | 0.11 | 1.02 (0.83, 1.26) | 0.847 |
|  |  |  | Weighted mode | -0.01 | 0.18 | 0.99 (0.70, 1.41) | 0.972 |
|  | Sutterella.id.2896 | 12 | Inverse variance weighted | 0.00 | 0.08 | 1.00 (0.86, 1.16) | 0.981 |
|  |  |  | MR Egger | 0.02 | 0.38 | 1.02 (0.49, 2.13) | 0.963 |
|  |  |  | Simple mode | 0.18 | 0.17 | 1.20 (0.86, 1.68) | 0.305 |
|  |  |  | Weighted median | 0.13 | 0.10 | 1.13 (0.93, 1.38) | 0.210 |
|  |  |  | Weighted mode | 0.17 | 0.17 | 1.19 (0.86, 1.65) | 0.319 |
|  | Terrisporobacter.id.11348 | 5 | Inverse variance weighted | 0.16 | 0.07 | 1.18 (1.02, 1.36) | 0.030 |
|  |  |  | MR Egger | 0.27 | 0.20 | 1.31 (0.88, 1.95) | 0.280 |
|  |  |  | Simple mode | 0.23 | 0.13 | 1.25 (0.97, 1.61) | 0.155 |
|  |  |  | Weighted median | 0.20 | 0.09 | 1.22 (1.01, 1.47) | 0.034 |
|  |  |  | Weighted mode | 0.22 | 0.13 | 1.24 (0.97, 1.59) | 0.163 |
|  | Turicibacter.id.2162 | 14 | Inverse variance weighted | -0.09 | 0.05 | 0.92 (0.83, 1.02) | 0.117 |
|  |  |  | MR Egger | 0.02 | 0.23 | 1.02 (0.65, 1.59) | 0.935 |
|  |  |  | Simple mode | 0.00 | 0.13 | 1.00 (0.78, 1.29) | 0.985 |
|  |  |  | Weighted median | -0.04 | 0.07 | 0.96 (0.83, 1.11) | 0.544 |
|  |  |  | Weighted mode | 0.00 | 0.13 | 1.00 (0.78, 1.28) | 1.000 |
|  | Tyzzerella3.id.11335 | 14 | Inverse variance weighted | -0.05 | 0.04 | 0.95 (0.88, 1.03) | 0.183 |
|  |  |  | MR Egger | 0.01 | 0.23 | 1.01 (0.65, 1.58) | 0.967 |
|  |  |  | Simple mode | -0.02 | 0.09 | 0.98 (0.83, 1.17) | 0839 |
|  |  |  | Weighted median | -0.03 | 0.05 | 0.97 (0.88, 1.08) | 0.608 |
|  |  |  | Weighted mode | -0.02 | 0.08 | 0.98 (0.83, 1.16) | 0.832 |
|  | unknowngenus.id.1000000073 | 16 | Inverse variance weighted | 0.00 | 0.07 | 1.00 (0.88, 1.15) | 0.968 |
|  |  |  | MR Egger | 0.18 | 0.19 | 1.19 (0.82, 1.74) | 0.372 |
|  |  |  | Simple mode | -0.01 | 0.15 | 0.99 (0.74, 1.32) | 0.944 |
|  |  |  | Weighted median | 0.01 | 0.08 | 1.01 (0.86, 1.17) | 0.939 |
|  |  |  | Weighted mode | -0.00 | 0.13 | 1.00 (0.78, 1.27) | 0.981 |
|  | unknowngenus.id.1000001215 | 12 | Inverse variance weighted | -0.03 | 0.06 | 0.97 (0.86, 1.10) | 0.656 |
|  |  |  | MR Egger | 0.31 | 0.16 | 1.36 (0.99, 1.87) | 0.088 |
|  |  |  | Simple mode | 0.07 | 0.14 | 1.07 (0.81, 1.42) | 0.626 |
|  |  |  | Weighted median | 0.02 | 0.07 | 1.02 (0.88, 1.18) | 0.837 |
|  |  |  | Weighted mode | 0.08 | 0.13 | 1.08 (0.84, 1.40) | 0.554 |
|  | unknowngenus.id.1000005472 | 15 | Inverse variance weighted | -0.02 | 0.06 | 0.98 (0.88, 109) | 0.701 |
|  |  |  | MR Egger | 0.14 | 0.18 | 1.15 (0.81, 1.63) | 0.440 |
|  |  |  | Simple mode | -0.06 | 0.12 | 0.94 (0.75, 1.19) | 0.636 |
|  |  |  | Weighted median | -0.03 | 0.08 | 0.97 (0.83, 1.12) | 0.661 |
|  |  |  | Weighted mode | -0.05 | 0.11 | 0.95 (0.77, 1.18) | 0.656 |
|  | unknowngenus.id.1000005479 | 10 | Inverse variance weighted | 0.05 | 0.06 | 1.05 (0.93, 1.18) | 0.411 |
|  |  |  | MR Egger | 0.52 | 0.29 | 1.68 (0.96, 2.97) | 0.109 |
|  |  |  | Simple mode | -0.06 | 0.13 | 0.94 (0.73, 1.21) | 0.649 |
|  |  |  | Weighted median | 0.03 | 0.08 | 1.03 (0.87, 1.20) | 0.751 |
|  |  |  | Weighted mode | -0.06 | 0.13 | 0.94 (0.73, 1.21) | 0.631 |
|  | unknowngenus.id.1000006162 | 17 | Inverse variance weighted | -0.01 | 0.04 | 0.99 (0.92, 1.07) | 0.827 |
|  |  |  | MR Egger | -0.08 | 0.17 | 0.92 (0.65, 1.30) | 0.647 |
|  |  |  | Simple mode | 0.02 | 0.10 | 1.02 (0.83, 1.25) | 0.839 |
|  |  |  | Weighted median | 0.00 | 0.05 | 1.00 (0.90, 1.11) | 0.994 |
|  |  |  | Weighted mode | -0.00 | 0.10 | 1.00 (0.83, 1.20) | 0.983 |
|  | unknowngenus.id.1868 | 13 | Inverse variance weighted | -0.04 | 0.06 | 0.96 (0.86, 1.07) | 0.480 |
|  |  |  | MR Egger | 0.14 | 0.16 | 1.15 (0.84, 1.59) | 0.397 |
|  |  |  | Simple mode | 0.09 | 0.12 | 1.09 (0.86, 1.39) | 0.487 |
|  |  |  | Weighted median | -0.04 | 0.08 | 0.96 (0.83, 1.12) | 0.609 |
|  |  |  | Weighted mode | -0.05 | 0.12 | 0.95 (0.75, 1.19) | 0.645 |
|  | unknowngenus.id.2001 | 11 | Inverse variance weighted | -0.04 | 0.06 | 0.96 (0.85, 1.09) | 0.548 |
|  |  |  | MR Egger | 0.04 | 0.22 | 1.04 (0.68, 1.59) | 0.849 |
|  |  |  | Simple mode | -0.04 | 0.12 | 0.96 (0.76, 1.20) | 0.707 |
|  |  |  | Weighted median | -0.03 | 0.08 | 0.97 (0.83, 1.15) | 0.757 |
|  |  |  | Weighted mode | -0.04 | 0.11 | 0.96 (0.77, 1.19) | 0.703 |
|  | unknowngenus.id.2041 | 12 | Inverse variance weighted | -0.04 | 0.05 | 0.96 (0.86, 1.06) | 0.416 |
|  |  |  | MR Egger | 0.26 | 0.16 | 1.29 (0.94, 1.77) | 0.141 |
|  |  |  | Simple mode | -0.04 | 0.14 | 0.96 (0.73, 1.27) | 0.801 |
|  |  |  | Weighted median | -0.02 | 0.07 | 0.98 (0.85, 1.13) | 0.773 |
|  |  |  | Weighted mode | 0.02 | 0.13 | 1.02 (0.79, 1.33) | 0.865 |
|  | unknowngenus.id.2071 | 18 | Inverse variance weighted | -0.04 | 0.06 | 0.96 (0.86, 1.08) | 0.523 |
|  |  |  | MR Egger | -0.16 | 0.29 | 0.85 (0.48, 1.51) | 0.596 |
|  |  |  | Simple mode | -0.10 | 0.15 | 0.90 (0.68, 1.20) | 0.499 |
|  |  |  | Weighted median | -0.08 | 0.07 | 0.93 (0.80, 1.07) | 0.308 |
|  |  |  | Weighted mode | -0.10 | 0.14 | 0.91 (0.69, 1.19) | 0.482 |
|  | unknowngenus.id.2755 | 15 | Inverse variance weighted | -0.04 | 0.05 | 0.96 (0.87, 1.06) | 0.399 |
|  |  |  | MR Egger | 0.05 | 0.21 | 1.05 (0.70, 1.57) | 0.828 |
|  |  |  | Simple mode | -0.03 | 0.12 | 0.97 (0.76, 1.23) | 0.808 |
|  |  |  | Weighted median | -0.03 | 0.07 | 0.97 (0.84, 1.12) | 0.671 |
|  |  |  | Weighted mode | -0.04 | 0.12 | 0.97 (0.76, 1.23) | 0.777 |
|  | unknowngenus.id.826 | 15 | Inverse variance weighted | -0.09 | 0.06 | 0.91 (0.81, 1.02) | 0.112 |
|  |  |  | MR Egger | -0.36 | 0.16 | 0.70 (0.51, 0.96) | 0.044 |
|  |  |  | Simple mode | 0.00 | 0.14 | 1.00 (0.76, 1.32) | 0.974 |
|  |  |  | Weighted median | -0.02 | 0.08 | 0.98 (0.84, 1.15) | 0.804 |
|  |  |  | Weighted mode | 0.01 | 0.17 | 1.01 (0.73, 1.40) | 0.955 |
|  | unknowngenus.id.959 | 13 | Inverse variance weighted | -0.02 | 0.04 | 0.98 (0.90, 1.06) | 0.572 |
|  |  |  | MR Egger | -0.09 | 0.26 | 0.92 (0.55, 1.53) | 0.749 |
|  |  |  | Simple mode | 0.04 | 0.09 | 1.04 (0.87, 1.24) | 0.666 |
|  |  |  | Weighted median | 0.02 | 0.06 | 1.02 (0.91, 1.14) | 0.746 |
|  |  |  | Weighted mode | 0.04 | 0.09 | 1.04 (0.88, 1.23) | 0.636 |
|  | Veillonella.id.2198 | 9 | Inverse variance weighted | -0.05 | 0.08 | 0.95 (0.82, 1.10) | 0.465 |
|  |  |  | MR Egger | 0.52 | 0.34 | 1.68 (0.86, 3.29) | 0.175 |
|  |  |  | Simple mode | -0.21 | 0.14 | 0.81 (0.61, 1.07) | 0.179 |
|  |  |  | Weighted median | -0.12 | 0.10 | 0.89 (0.74, 1.07) | 0.225 |
|  |  |  | Weighted mode | -0.20 | 0.15 | 0.82 (0.61, 1.09) | 0.214 |
|  | Victivallis.id.2256 | 12 | Inverse variance weighted | 0.04 | 0.04 | 1.04 (0.97, 1.11) | 0.290 |
|  |  |  | MR Egger | -0.38 | 0.23 | 0.69 (0.44, 1.08) | 0.135 |
|  |  |  | Simple mode | 0.06 | 0.07 | 1.06 (0.92, 1.22) | 0.452 |
|  |  |  | Weighted median | 0.06 | 0.05 | 1.06 (0.97, 1.16) | 0.212 |
|  |  |  | Weighted mode | 0.05 | 0.08 | 1.05 (0.90, 1.23) | 0.512 |

Significant (p < 0.05) associations estimated via inverse-variance-weighted Mendelian randomization are shown in green for a reduction in sepsis risk and red for an increase in sepsis risk due to increased abundance of gut microbiota.
