# Supplementary material for: Causal effects of gut microbiota on sepsis: a two-sample Mendelian randomization study
Source: Front Microbiol. 2023 May 10;14:1167416. doi: 10.3389/fmicb.2023.1167416 (PMC10206031; doi:10.3389/fmicb.2023.1167416)

Supplementary Figures

**Supplementary Figure S1** Forest plots of the causal effects of gut microbiota on the risk of sepsis.

(A)class *Deltaproteobacteria*; (B)order *Desulfovibrionales*; (C)family *Clostridiaceae1*; (D)genus *Alloprevotella*; (E)genus *Catenibacterium*; (F)genus *Hungatella*; (G)genus *LachnospiraceaeND3007group*; (H)genus *Terrisporobacter*.


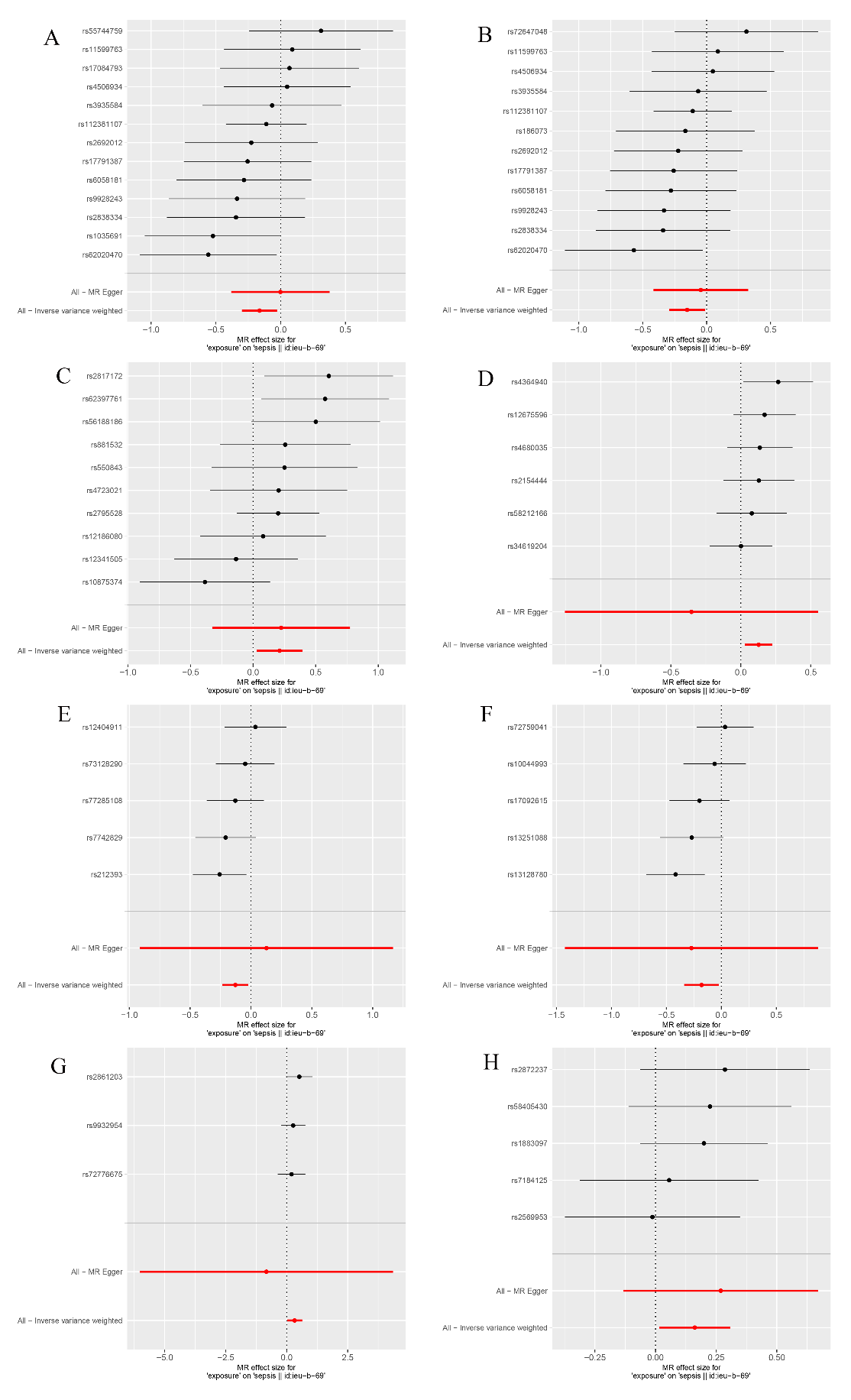


**Supplementary Figure S2** Leave-one-out sensitivity analyses of the causal effects of gut microbiota on the risk of sepsis.

(A)class *Deltaproteobacteria*; (B)order *Desulfovibrionales*; (C)family *Clostridiaceae1*; (D)genus *Alloprevotella*; (E)genus *Catenibacterium*; (F)genus *Hungatella*; (G)genus *LachnospiraceaeND3007group*; (H)genus *Terrisporobacter*.


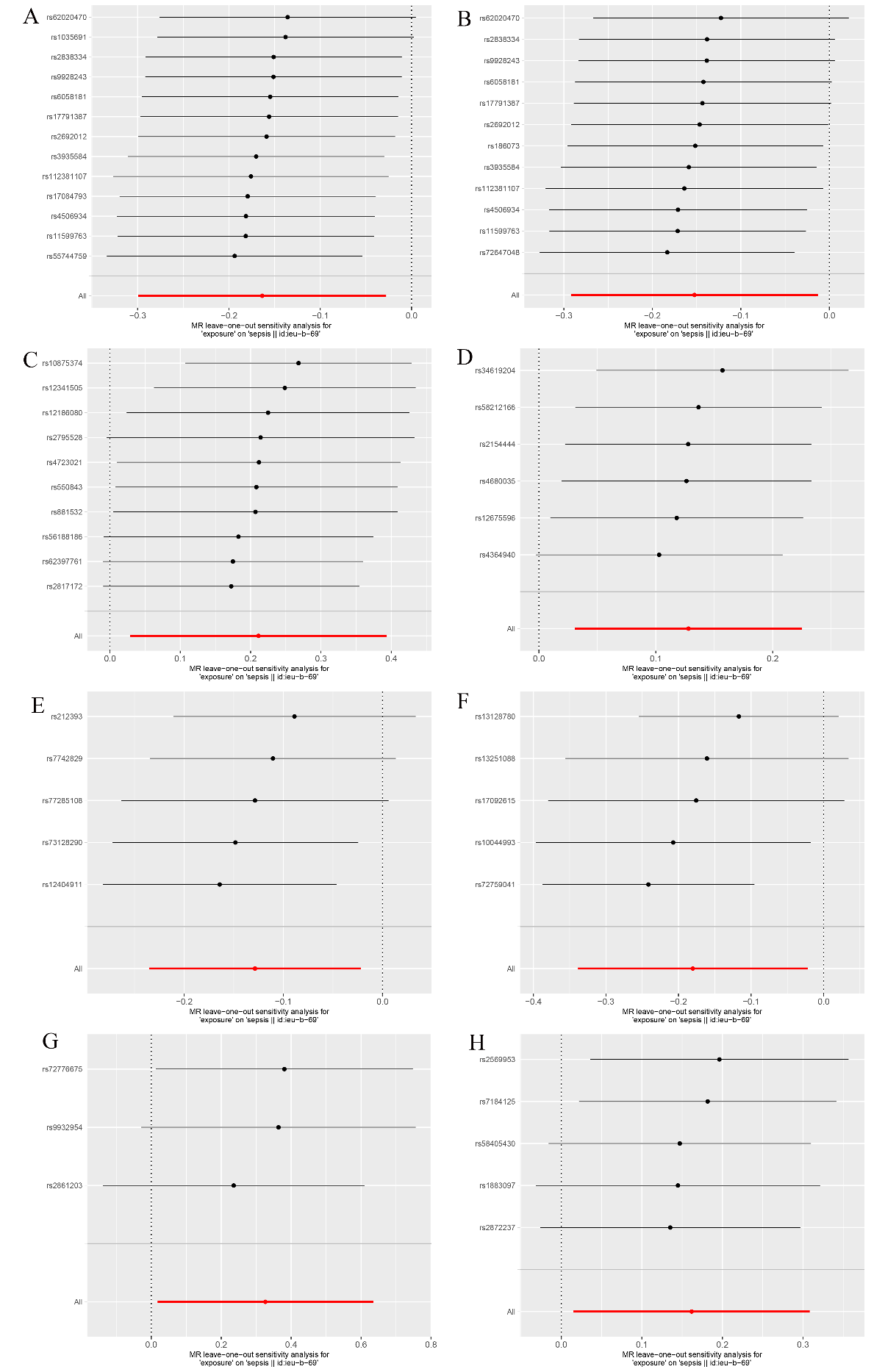

Supplement: Supplementary file 3 [file Data_Sheet_1.docx]
